# West Nile virus and Zika virus infections induce aggresome formation in human neural progenitor and A549 cells

**DOI:** 10.1128/jvi.02080-25

**Published:** 2026-05-11

**Authors:** Mausumi Basu, Emilio E. Espínola, Weibo Niu, Zhexing Wen, Margo A. Brinton

**Affiliations:** 1 Department of Biology, Georgia State University 123426, Atlanta, Georgia, USA; 2 Department of Psychiatry and Behavioral Sciences, Emory University School of Medicine 12239, Atlanta, Georgia, USA; 3 Department of Cell Biology, Emory University School of Medicine 12239, Atlanta, Georgia, USA; 4 Department of Neurology, Emory University School of Medicine 12239, Atlanta, Georgia, USA; 5 Department of Human Genetics, Emory University School of Medicine 12239, Atlanta, Georgia, USA; University of Kentucky College of Medicine, Lexington, Kentucky, USA

**Keywords:** West Nile virus (WNV), Zika virus (ZIKV), asymmetric replication vesicles, h-NPCs, A549 cells, GRP78, HDAC6, aggresome, acetylated/deacetylated tubulin, vimentin

## Abstract

Zika viral replication vesicles (VRVs) were previously observed to cluster asymmetrically on one side of the nucleus in infected Huh7, A549, and h-NPCs. West Nile virus (WNV) infections in these cells also induce asymmetrically clustered VRVs. The sequential cell responses leading to asymmetric clustering of the WNV and ZIKV VRVs were investigated. Both infections activated the cellular unfolded protein response (UPR), as indicated by increased expression of GRP78, the master regulator of UPR, and activation of the PERK branch of the UPR (p-PERK–p-eIF2α–GADD34). Increased microtubule (MT) acetylation was also induced. As the infection progressed, the level of ubiquitinated proteins increased and GRP78, ubiquitinated proteins, the deacetylase HDAC6, VRVs, and modified endoplasmic reticulum clustered at the cellular microtubule-organizing center (MTOC) and became surrounded by a vimentin “cage.” The components involved in the formation of asymmetric VRVs are characteristic of aggresome formation, an additional cellular response that copes with excessive misfolded proteins when the UPR is overwhelmed. During aggresome formation, HDAC6 links polyubiquitinated proteins to the dynein motor complex for transport along MTs to the MTOC, where they aggregate and are surrounded by a vimentin cage. HDAC6 knockdown prevented asymmetric clustering of viral replication complexes, resulted in activation of the p-PERK–p-eIF2α–CHOP apoptotic pathway, and decreased viral yield. The threshold for triggering aggresome formation varied with the infecting orthoflavivirus and cell type. The formation of aggresome-like, asymmetric VRVs in A549 cells and h-NPCs infected with WNV or ZIKV delayed activation of the cellular death pathways and increased viral yield.

**IMPORTANCE:**

Zika virus and West Nile virus replication complexes, which are typically symmetrically distributed around the nucleus in the perinuclear region of an infected cell, cluster asymmetrically on one side of the nucleus in A549 and human neural progenitor cells as the infections progress. Analysis of the sequential cellular responses involved showed that microtubule acetylation increased and the unfolded protein response was activated; then, the levels of ubiquitinated protein and HDAC6 increased, followed by asymmetric concentration of the viral replication complexes, ubiquitinated proteins, and HDAC6 at the cellular microtubule-organizing center, where they became surrounded by a vimentin cage. These sequential steps are characteristic of aggresome formation, an additional protective cellular response that copes with the accumulation of toxic misfolded proteins. This response delayed activation of cell death pathways and extended virus production. Both cell type-specific and orthoflavivirus-specific characteristics determine whether a particular infection induces aggresome formation.

## INTRODUCTION

The mosquito-borne viruses, West Nile virus (WNV) and Zika virus (ZIKV), are members of the genus *Orthoflavivirus* in the family *Flaviviridae*. WNV is maintained in nature in a bird-mosquito-bird transmission cycle. Humans are “dead end” hosts for WNV infections due to low virus levels in their blood that are not sufficient to infect mosquitoes. Most human WNV infections are asymptomatic, but some people develop a mild febrile illness, and ~1% progress to severe central nervous system disease that can be fatal (1). ZIKV is primarily transmitted to humans by mosquitoes, but sexual transmission can occur (2, 3). A ZIKV infection in a fetus *in utero* can result in delivery of a child with congenital microcephaly or neurodevelopmental abnormalities, with first-trimester infections posing the greatest risk for these outcomes (4). A few adults develop encephalitis or Guillain-Barré syndrome after infection with ZIKV (5).

The orthoflavivirus genome is a single-stranded, positive-sense RNA of ~11 kb that encodes a single open reading frame and has a 5′ Type 1 cap but does not have a 3′ terminal poly A. Virions attach to yet unknown cellular receptors and enter cells by endocytosis (6). The genome RNA is released into the cytoplasm following acid-mediated fusion of the virion and endosomal membranes. A single polyprotein is translated from the genome on the cellular rough endoplasmic reticular (ER) membrane and then cleaved by viral and cellular proteases into the mature structural proteins (capsid, PreM, and E) and nonstructural proteins (NS1, NS2A, NS2B, NS3, NS4A, NS4B, and NS5). The genome RNA switches from translation to replication after it cyclizes through 3′–5′ RNA-RNA interactions. The viral polymerase NS5 interacts with the 5′ terminal genome RNA structure and then initiates synthesis of a single minus strand from the 3′ end of the cyclized genome RNA generating a dsRNA. The minus strand in this dsRNA is then used as the template for nascent genome RNA synthesis. Genome RNA synthesis initially occurs at a low level in infected cells. As the infection progresses, invaginations in the ER membrane, named viral replication vesicles (VRVs), are induced by viral proteins working with cellular proteins. The VRVs contain viral nonstructural protein replication complexes, viral dsRNA, and some host proteins. Exponential genome synthesis occurs in the VRVs. Most of the nascent genome RNAs exiting through a VRV pore become associated with the rough ER and are translated. A few of the released nascent genome RNAs interact with nearby regions of the ER membrane that have capsid dimers attached to their cytoplasmic side and inserted E and pre-M proteins. This interaction results in concurrent virion assembly and budding into the ER lumen. The newly formed virions are then transported through the host cell’s secretory pathway and ultimately released from the cell via exocytosis, completing the viral life cycle.

Orthoflavivirus polyprotein translation, exponential genome synthesis, and virion assembly each occur on infection-modified ER membranes that together have been termed the viral replication factory (7–9). One hundred and eighty genome translations are required to produce the structural proteins for a single nascent virion, and each infected cell produces thousands of virions. The greater the efficiency of infection, the more viral protein that is produced, which increases the likelihood that the capacity of the infected cell to maintain ER homeostasis will be overwhelmed. In addition, the very large orthoflaviviral polyprotein, which contains multiple transmembrane regions, imposes stress on the cellular translation quality control pathways (10).

It was previously shown that orthoflavivirus infections can induce ER stress and activate the unfolded protein response (UPR) (10–12). Within cells in a state of ER homeostasis, the UPR master regulator, GRP78 (also known as Bip), is bound to the three UPR transmembrane sensors, ATF6, PERK, and IRE1, and maintains them in an inactive state (13). The accumulation of unfolded/misfolded proteins in the lumen of the ER induces ER stress. In response to ER stress, GRP78 protein levels typically increase. GRP78 binds to unfolded and misfolded proteins in the ER lumen, aiding their proper folding and preventing protein aggregation. The sensors are activated when UPR sensor bound GRP78 is released, but the three sensors can be activated to different levels (13–15). After the release of GRP78, the PERK sensor dimerizes and is activated by autophosphorylation. Activated PERK phosphorylates the eukaryotic translation-initiation factor 2α (eIF2α), which attenuates cellular translation initiation through stress granule formation (16). Phosphorylated eIF2α also selectively enhances the translation of ATF4 mRNA. ATF4 enters the nucleus and activates the expression of its key target genes, PPP1R15A (GADD34) and DDIT3 (CHOP) (17). GADD34 is a regulatory subunit of protein phosphatase 1 (PP1) that dephosphorylates p-eIF2α allowing recovery of translation (18). Although the expression of CHOP mRNA is rapidly upregulated by an ER stress response, the second ORF of this mRNA, which encodes CHOP, is translated only when a cell is unable to restore homeostasis due to severe and/or prolonged ER stress (19). CHOP activates apoptotic gene expression (20). An aggresome is a perinuclear, membrane-free structure that serves as a cellular quality-control compartment where misfolded or aggregated proteins are sequestered. Aggresome formation was first observed in cells overexpressing recombinant proteins (21, 22). This cellular response is triggered when the accumulation of ubiquitinated misfolded proteins in the ER lumen exceeds the capacity of the chaperone refolding system or other cellular clearance mechanisms becomes overwhelmed. Aggresome formation was first observed in cells overexpressing recombinant proteins (21, 22). Histone deacetylase 6 (HDAC6), a key protein involved in aggresome formation, links polyubiquitinated proteins to the dynein motor complex for transport along MTs to the microtubule-organizing center (MTOC), where the aggregates become surrounded by a vimentin cage (21, 23, 24).

Previous studies found that ZIKV infections induced asymmetric VRVs clustered at the MTOC in A549 and human neural progenitor cells (h-NPCs), with the asymmetric VRVs and the nucleus surrounded by an MT cage (9, 25). The data obtained in the present study indicate that WNV infections in A549 cells and h-NPCs also induce the formation of asymmetrically clustered VRVs at the MTOC. RNA-seq data indicated that both ZIKV and WNV infections upregulated the expression of multiple UPR genes, as well as of HDAC6 and ubiquitin enzyme genes, and increased the levels of GRP78, p-PERK, and GADD34 proteins. The sequential steps involved in aggresome formation, namely the aggregation and ubiquitination of accumulated misfolded proteins, their association with HDAC6, followed by HDAC6-mediated trafficking on MTs to the MTOC, where the clustered aggregates are surrounded by a vimentin cage, were found to be required for the formation of asymmetrically concentrated orthoflavivirus VRVs at the MTOC. Depolymerization of MTs or HDAC6 knockdown inhibited the formation of asymmetric VRVs. The asymmetric VRVs were stained with the aggresome detection dye Proteostat. An unacetylated MT cage surrounded the vimentin-caged VRVs and the nucleus, likely due to the high concentration of HDAC6 at the MTOC. The formation of aggresome-like, asymmetric VRVs in A549 cells and h-NPCs infected with WNV or ZIKV delayed the activation of death pathways and extended virus production.

## RESULTS

### A WNV infection induces asymmetric clustering of viral replication vesicles (VRVs) in A549 cells

**Fig 1:**
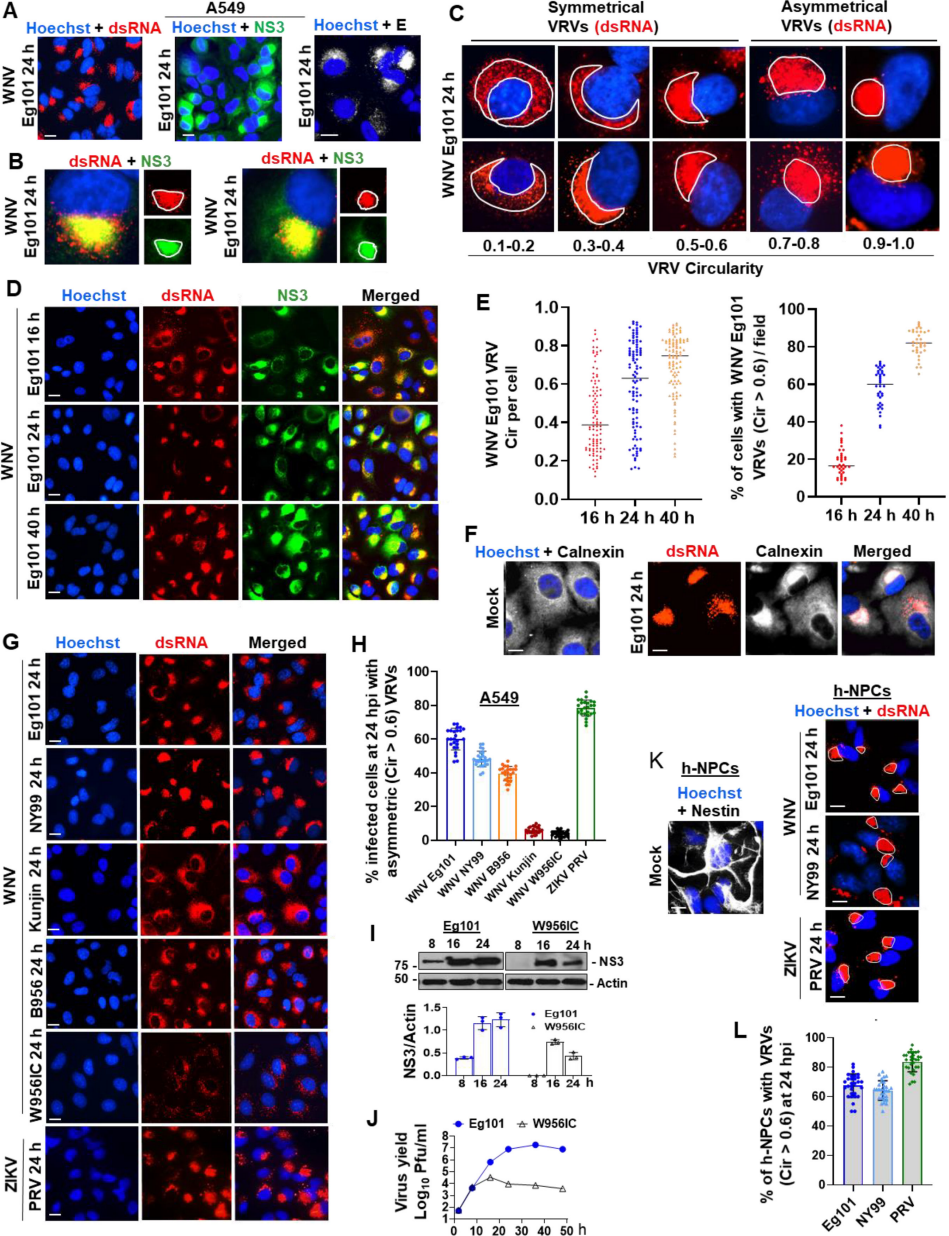
WNV infections induce asymmetric localization of the VRVs. (**A**) A549 cells grown to 70–80% confluency on coverslips in 24-well plates were infected with WNV Eg101 at a MOI of 1. At 24 hpi, the cells were fixed, permeabilized, and processed for IFA. VRVs were detected by anti-dsRNA (red) or anti-NS3 (green) or anti-E antibody (white), and nuclei were stained with Hoechst 33258 dye (blue). (**B**) WNV Eg101-infected A549 cells (MOI 1) were immuno-stained with anti-dsRNA (red) and anti-NS3 (green) antibody at 24 hpi. (**C**) The circularity (Cir) of the VRV distribution area (surrounded by a white line) in individual cells was calculated using the formula 4π (area/perimeter^2^). A perfect circle was considered to have a value of 1. (**D**) WNV Eg101-infected A549 cells (MOI 1) were processed for IFA at 16, 24, and 40 hpi. VRVs were detected by anti-dsRNA (red) and anti-NS3 (green) antibody. (**E**) Quantification of VRV Cir per cell and percentage of WNV-infected cells with VRV Cir >0.6/field at different hpi. (**F**) Mock- and WNV Eg101-infected A549 cells (MOI 1) were processed for IFA at 24 hpi to detect ER using anti-calnexin antibody (white) and VRVs with anti-dsRNA (red) antibody (**G**) IFA of A549 cells infected with WNV Eg101, NY99, Kunjin, B956, the WNV chimera W956IC, or ZIKV PRVABC59 (PRV) (MOI 1) were processed for IFA at 24 hpi with anti-dsRNA (red) antibody. (**H**) Quantification of the percentage of infected cells with VRVs of Cir >0.6. (**I**) A549 cells were infected with WNV Eg101 or W956IC (MOI of 1). Viral NS3 levels were detected in cell lysates by western blotting, and (**J**) virus yields were determined by plaque assay. (**K**) h-NPCs were mock-infected or infected with WNV Eg101 or NY99 or ZIKV PRV and processed for IFA. VRVs were detected by anti-dsRNA antibody (red) and h-NPCs by anti-nestin (purple) antibody. (**L**) Percentage of infected h-NPCs with VRVs of Cir >0.6 at 24 hpi. In this and all subsequent figures, representative images are shown. IFA quantification data were obtained from three biological experiments. IFA images were acquired with an Axio Observer Z1 (Zeiss) wide field fluorescence microscope using a 63× oil immersion objective and processed for quantification with Volocity 6.0.1 or ImageJ software. Scale bars: 60 µm (µm/pixel [X] and µm/pixel [Y] were adjusted to 1].

The VRVs detected by an immunofluorescence assay (IFA) using either anti-dsRNA, anti-NS3, or anti-E antibody in our initial study of a WNV Eg101 infection in A549 cells were observed to cluster asymmetrically in most infected cells by 24 h post-infection (hpi) (Fig. 1A and B). The shape factor or circularity (Cir) of the perinuclear area in which most of the VRVs were distributed in individual WNV Eg101-infected A549 cells was measured using Image J software. A perfect circle has a Cir value of 1.0. The shape of a cytoplasmic area containing VRVs that were distributed symmetrically around the nucleus had a Cir value less than or equal to 0.6 (Cir ≤ 0.6), while the shape of an area containing VRVs asymmetrically concentrated on one side of the nucleus had a Cir value greater than 0.6 (Cir > 0.6) (Fig. 1C). NS3 or dsRNA foci that were located outside the perinuclear region were not included in the assessed areas (Fig. 1B and C). Measurement of the Cir value of the distribution area of most of the VRVs in individual Eg101-infected cells at different times after infection indicated that the VRVs were distributed asymmetrically (Cir >0.6) in ~16% of the infected cells by 16 hpi, ~65% by 24 hpi, and ~80% by 40 hpi (Fig. 1D and E). The data indicated an increase in the number of infected cells with asymmetric VRVs as the infection progressed. Orthoflavivirus translation, RNA replication, and virion assembly occur in close association with the ER. The cellular distribution of the ER in WNV-Eg101-infected A549 cells was assessed by IFA using anti-calnexin antibody. In mock-infected cells, calnexin was distributed symmetrically around the nucleus (Fig. 1F). By 24 hpi in WNV Eg101-infected cells, some ER had concentrated and colocalized with the asymmetric VRVs. A Kunjin virus infection was previously shown not to induce the formation of asymmetric VRVs in A549 cells (25). To determine whether infections with other WNV strains in addition to Eg101 induced asymmetric VRVs, A549 cells were infected with either the lineage I WNV strains Eg101, NY99, or Kunjin (Kun); the lineage II WNV strain B956; the lineage II/lineage I chimeric WNV W956IC; or Zika virus PRVABC59 (ZIKV PRV) (Fig. 1G). By 24 hpi, ~60% of the WNV Eg101, ~50% of the WNV NY99, ~40% of the WNV B956, ~ 7% of the WNV Kun, ~2% of the WNV W956IC, and ~ 80% of ZIKV PRV-infected A549 cells contained asymmetric VRVs (Fig. 1H). Infections with the WNV lineage I strains Eg101 or NY99 or the lineage II strain B956 efficiently induced asymmetric VRVs by 24 hpi, whereas Kun and W956IC infections did not. The ZIKV PRV infection was the most efficient at inducing asymmetric VRVs.

Viral NS3 levels were assessed in cellular extracts harvested from W956IC- and Eg101-infected A549 cells at different times after infection by western blotting (Fig. 1I). Viral protein levels increased by 8 hpi and were maintained at a high level through 24 hpi in the WNV Eg101-infected cells. In contrast, in the W956IC-infected cells, the viral protein levels increased to a lower level by 8 and 16 hpi and decreased by 24 hpi. The virus yield produced by the WNV Eg101-infected cells was ~10^7^ PFU/mL by 24 hpi and remained high through 48 hpi, whereas the yield from the W956IC-infected cells did not increase above ~10^4^ PFU/mL (Fig. 1J). We previously showed that a W956IC infection is unique in preferentially amplifying viral RNA rather than viral protein at early times after infection in A549 cells, which results in robust activation of the innate immune response and limits virus replication and virion production (26). It was previously shown that a WNV Kun infection produced less viral protein and a lower viral yield than a WNV NY99 infection due to a higher sensitivity to type 1 interferon (IFN) (27). By 24 hpi, asymmetrically clustered VRVs (Cir > 0.6) were observed in ~70% of WNV Eg101, in ~65% of WNV NY99, and in ~85% of ZIKV PRV-infected h-NPCs (Fig. 1K and L).

### WNV and ZIKV infections activate the UPR in A549 cells

**Fig 2:**
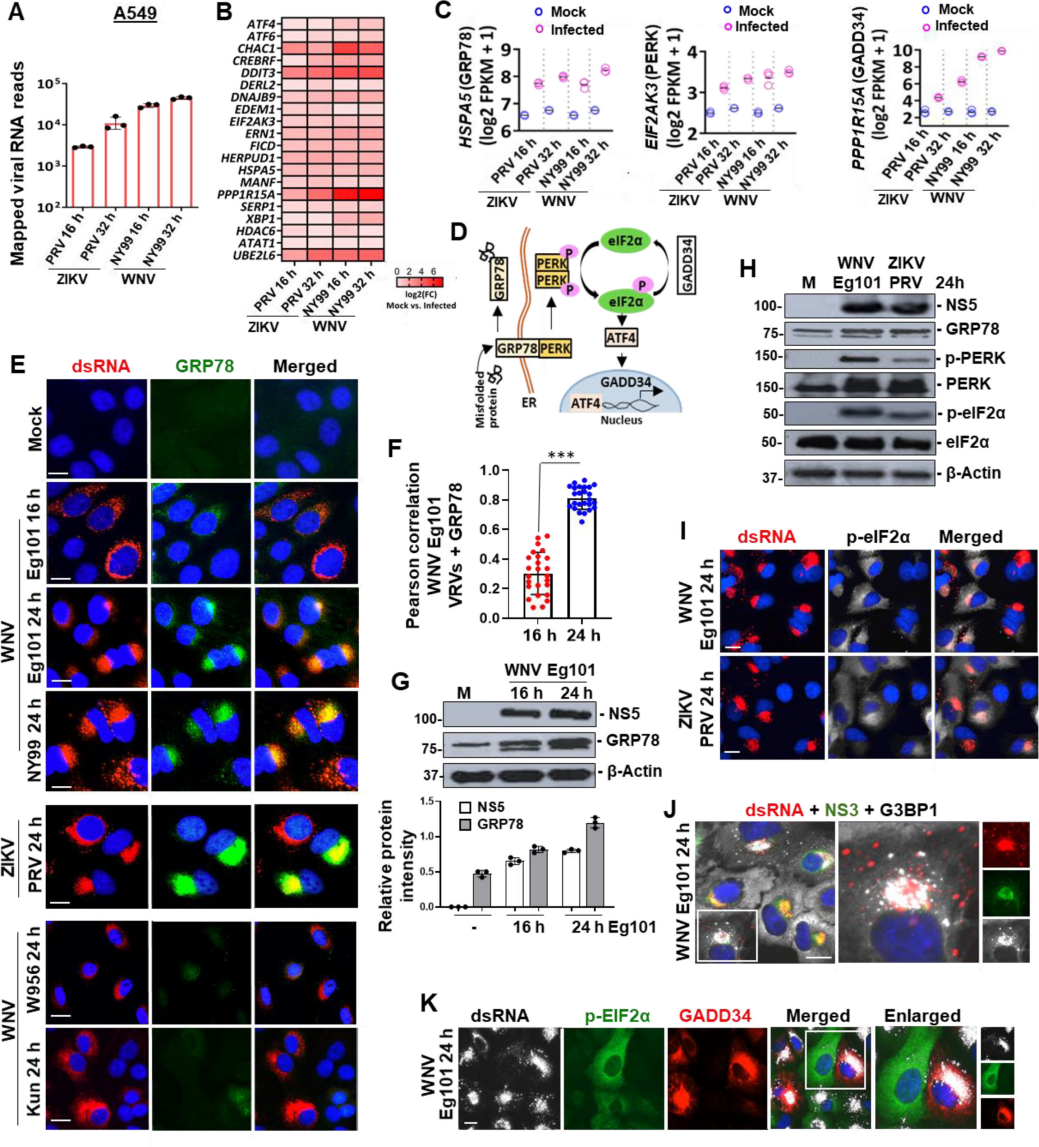
ZIKV and WNV infections activate the UPR in A549 cells. (**A**) Relative intracellular viral RNA levels determined by RNAseq analysis in A549 cells infected with WNV NY99 or ZIKV PRV (MOI of 1) for 16 or 32 h. (**B**) UPR pathway genes upregulated by the WNV and ZIKV infections. (**C**) HSPA5 (GRP78), EIF2AK3 (PERK), and PPP1R15A (GADD34) mRNA expression in WNV NY99 and ZIKV PRV-infected A549 cells by 16 and 32 hpi. (**D**) Diagram of PERK pathway activation. (**E**) A549 cells were mock-infected or infected with WNV Eg101 or WNV NY99 or WNV Kun or W956 IC or ZIKV PRV (MOI of 3) and processed for IFA at the indicated times to GRP78 (green) and VRVs (dsRNA, red). (**F**) Pearson correlation coefficient data indicating the extent of colocalization of VRVs and GRP78 in WNV Eg101-infected cells at 16 and 24 hpi. (**G**) Western blot analysis of cell lysates harvested from mock- or WNV Eg101 (MOI 1)-infected A549 cells at 16 and 24 hpi. The NS5 and GRP78 band intensities were quantified and normalized to β-actin. (**H**) Western blot analysis of p-PERK and p-eIF2α in WNV Eg101- or ZIKV PRV-infected (MOI 1) A549 cell lysates at 24 hpi. (**I**) A549 cells infected with WNV Eg101 or ZIKV PRV (MOI 3) were processed for IFA at 24 hpi to detect p-eIF2α (white) and VRVs (dsRNA, red). (**J**) WNV Eg101-infected (MOI 3) A549 cells were processed for IFA at 24 hpi to detect G3BP1 (SG marker, white), dsRNA (red), and NS3 (green). The area in the white box was enlarged. (**K**) A549 cells infected with WNV Eg101 (MOI of 3) were processed for IFA to detect GADD34 (red), p-eIF2α (green), and VRVs (dsRNA, white). Scale bars: 60 µm (µm/pixel [X] and µm/pixel [Y] were adjusted to 1). Statistical significance was determined by a Student’s *t-*test. ***P < 0.001.

The data obtained suggested an association between higher intracellular viral protein levels and the induction of asymmetric VRVs. A transcriptome analysis was used as an initial means of determining whether a UPR response was induced by the viral infections that promoted the formation of asymmetric VRVs. Total cellular RNA was extracted from WNV NY99- or ZIKV PRV-infected A549 cells at 16 and 32 hpi and was subjected to RNA-seq analysis. The RNA was polyA-selected prior to library construction. Although orthoflavivirus genomes do not have a 3′ poly-(A) tail, viral RNA reads were detected and mapped to the respective viral genome sequences to obtain approximate estimates of the viral RNA levels present. Higher intracellular viral RNA levels were detected in the WNV-infected cells than in the ZIKV-infected cells (Fig. 2A; Table S1). Transcriptomic and gene ontology analyses of the RNA-seq data revealed upregulation of the expression of multiple UPR genes, including *HSPA5* (GRP78), the three UPR sensors *EIF2AK3* (PERK), *ERN1* (IRE1), and *ATF6*, *PPP1R15A* (GADD34), and *DDIT3* (CHOP), as well as *UBE2L6* (ubiquitin/ISG15-conjugating enzyme E2 L6), by both the ZIKV and WNV infections in A549 cells (Fig. 2B; Table S2). By 16 hpi, the expression of GRP78, PERK, and GADD34 mRNA was strongly upregulated by both the WNV NY99 and ZIKV PRV infections in three replicate samples for each infection and further increased by 32 hpi (Fig. 2C; Table S3). Downstream effects of the PERK sensor activation are illustrated in Fig. 2D. Intracellular GRP78 protein levels were assessed by IFA in A549 cells that were mock-infected or infected with WNV Eg101, WNV NY99, WNV Kun, W956IC, or ZIKV PRV. In mock-infected cells, GRP78 (faint signal) was diffusely distributed in the cytoplasm (Fig. 2E). At 16 hpi, WNV Eg101-infected cells with symmetrical VRVs contained a faint cytoplasmic GRP78 signal, but by 24 hpi, the GRP78 signal intensity had increased, and most of the GRP78 colocalized with the asymmetric VRVs. Pearson correlation coefficient data confirmed positive colocalization of dsRNA and GRP78 (Fig. 2F). Colocalization of a GRP78 signal of increased intensity with asymmetric VRVs was also observed in WNV NY99- and ZIKV PRV-infected cells by 24 hpi. In contrast, at 24 hpi, 95% of the W956IC- or Kun-infected cells contained a dispersed, faint GRP78 signal, and the VRVs were symmetrically distributed in the perinuclear area (Fig. 2E). A western blot analysis indicated that GRP78 protein levels increased with time after a WNV Eg101 infection in A549 cells (Fig. 2G). Increasing intercellular GRP78 protein levels are characteristic of UPR activation (15). Cell lysates harvested from WNV Eg101- and ZIKV PRV-infected A549 cells at 24 hpi were next analyzed for PERK and eIF2α phosphorylation by western blotting. Phosphorylated PERK and eIF2α were detected in lysates from WNV- and ZIKV-infected cells by 24 hpi (Fig. 2H). An IFA detected p-eIF2α in 30–40% of WNV Eg101- and ZIKV PRV-infected A549 cells by 24 hpi, with the intensity of the p-eIF2α signal varying among infected cells (Fig. 4I). p-eIF2α reduces global protein synthesis through stress granule (SG) formation and upregulates the production of proteins that help restore cellular homeostasis (28). IFA detection of the SG marker G3BP1 detected SG formation in ~30% of the WNV-infected cells by 24 hpi (Fig. 2J). Lower p-eIF2α and higher dsRNA signal intensities were detected in infected cells expressing GADD34 due to eIF2α dephosphorylation (Fig. 2K). These data indicated that the UPR and its downstream target genes were activated in cells with asymmetric VRVs.

### Asymmetrically clustered VRVs have aggresome characteristics

**Fig 3:**
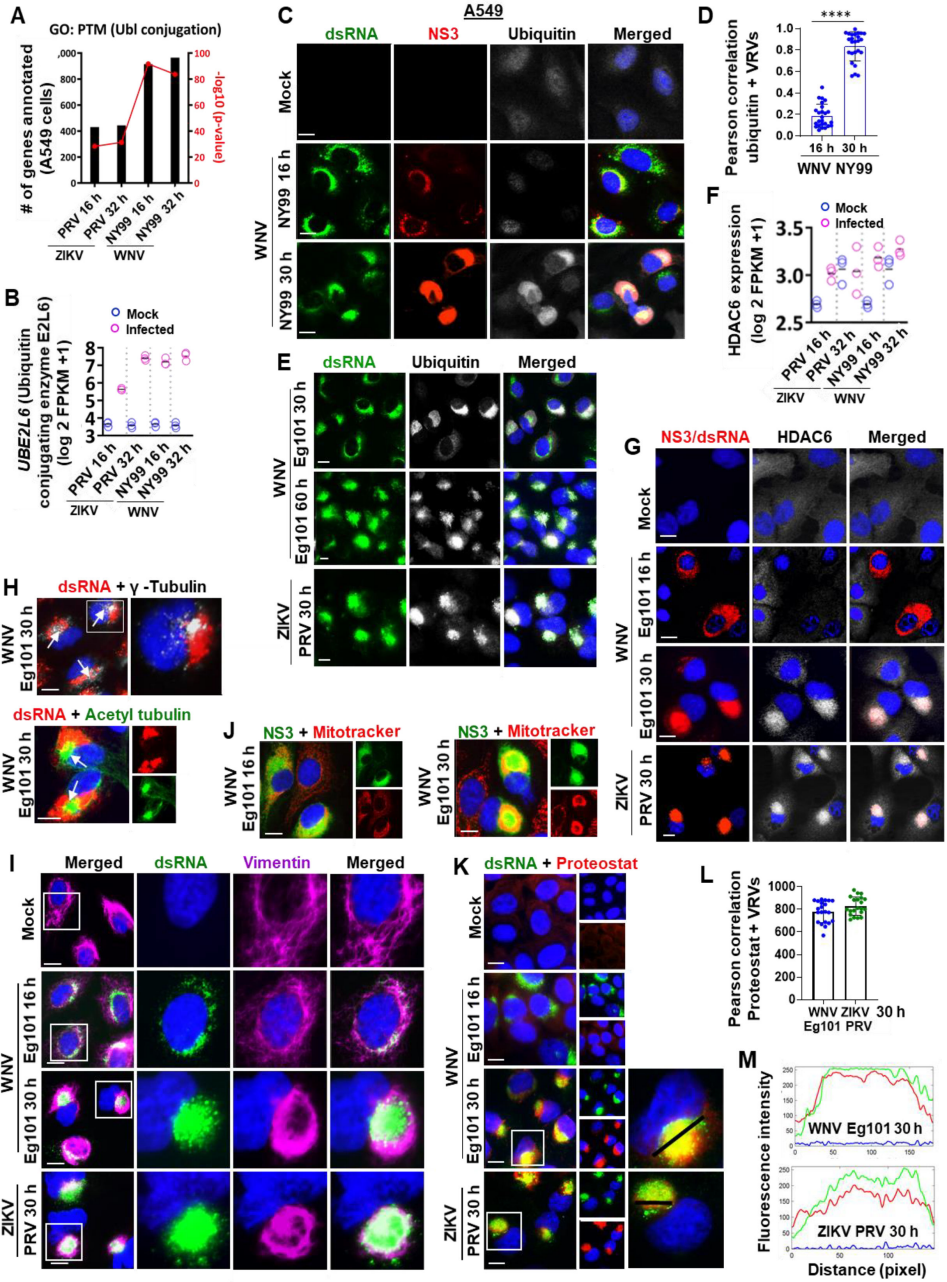
Aggresome components colocalize with asymmetric VRVs. (**A**) Number of upregulated genes encoding a protein that can be post-translationally modified by at least one ubiquitin-like modifier protein in A549 cells infected with WNV NY99 or ZIKV PRV (MOI of 1). (**B**) *UBE2L6* mRNA expression in WNV NY99 or ZIKV PRV-infected A549 cells by 16 and 32 hpi. (**C**) Mock- or WNV NY99-infected A549 cells (MOI 3) were processed for IFA at 16 or 30 hpi to detect ubiquitin (white), NS3 (red), and dsRNA (green). (**D**) Pearson correlation coefficient/cell indicating the extent of colocalization of VRVs and ubiquitinated protein in WNV NY99-infected cells at 16 and 30 hpi. (**E**) A549 cells infected with WNV Eg101 or ZIKV PRV (MOI 3) were processed for IFA at the indicated times to detect ubiquitin (white) and dsRNA (green). (**F**) *HDAC6* mRNA expression in A549 cells infected with WNV NY99 or ZIKV PRV at 16 or 32 hpi. (**G**) A549 cells mock-infected or infected with WNV Eg101 or ZIKV PRV were processed for IFA to detect HDAC6 (white). VRVs were detected with anti-NS3 or anti-dsRNA (red) antibody. (**H**) Detection of the MTOC (indicated by a white arrow) in WNV Eg101-infected A549 cells using anti-γ-tubulin (white) and anti-acetyl tubulin (green) antibodies. (**I**) A549 cells mock-infected or infected with WNV Eg101 or ZIKV PRV (MOI of 3) were processed for IFA to detect vimentin (purple). (**J**) Mock-infected or WNV Eg101-infected A549 cells (MOI of 3) were stained with Red MitoTracker to detect mitochondria at 16 or 30 hpi. VRVs were detected by anti-NS3 antibody (green). (**K**) Mock-infected and WNV Eg101- or ZIKV PRV-infected (MOI 3) A549 cells were stained with Proteostat (aggresome detection red dye) and processed for IFA. VRVs were detected with anti-dsRNA (green) antibody. (**L**) Pearson correlation coefficient/cell data. (**M**) Fluorescence intensity profile plots of Proteostat and VRV colocalization across the indicated area (black line) in WNV Eg101- and ZIKV PRV-infected A549 cells at 30 hpi. Scale bars, 60 µm (µm/pixel [X] and µm/pixel [Y] were adjusted to 1). Statistical significance was determined by a Student’s *t-*test. ****P < 0.0001.

Once a threshold concentration of accumulated unfolded and/or misfolded proteins within the ER lumen is reached, stressed cells respond by increasing unfolded/misfolded protein ubiquitination, and the ubiquitinated, unfolded/misfolded proteins then aggregate due to hydrophobic interactions (21, 29). The transcriptomic data were queried to determine the number of cellular genes upregulated by WNV or ZIKV infection in A549 cells that encode proteins that can be modified post-transcriptionally by the addition of ubiquitin-like proteins. Both virus infections upregulated genes in this class, with the WNV infection upregulating a higher number of these genes (Fig. 3A; Table S4). The expression of *UBE2L6*, which encodes a ubiquitin-conjugating enzyme, was strongly upregulated by both infections (Fig. 3B; Table S3). The signal intensity of ubiquitinated proteins detected by IFA using an anti-ubiquitin antibody was low and diffuse in mock-infected and in WNV NY99-infected cells at 16 hpi. (Fig. 3C). By 30 hpi, much of the ubiquitinated protein detected colocalized with the asymmetric VRVs in cells infected with WNV NY99, WNV Eg101, or ZIKV PRV (Fig. 3C through E). By 60 hpi, the asymmetrically located ubiquitin signal intensity remained strong in 100% of the WNV Eg101-infected cells, indicating that the ubiquitinated protein aggregates had not been degraded.

Ubiquitinated protein aggregates that interact with the C-terminal ubiquitin-binding domain of the MT-associated cytoplasmic histone deacetylase 6 (HDAC6) are delivered to the perinuclear region near the MTOC by dynein-dependent retrograde transport on the MTs, forming an aggresome (23). HDAC6 mRNA expression was upregulated by both the WNV NY99 and ZIKV PRV infections in each of the three replicate samples by 16 hpi (Fig. 3F; Table S3). Diffuse cytoplasmic HDAC6 was detected in mock-infected and WNV Eg101-infected A549 cells with VRVs Cir ≤ 0.6 by 16 hpi. By 30 hpi, HDAC6 signal intensity had increased and colocalized with the asymmetric VRVs (Cir > 0.6) in WNV Eg101- and ZIKV PRV-infected cells (Fig. 3G). The MTOC was detected by IFA using an anti-γ-tubulin antibody and was observed as a bright spot on one side of the nucleus that colocalized with the asymmetric VRVs (Fig. 3H). An IFA using an anti-acetyl tubulin antibody detected concentrated acetylated MTs, characteristic of the MTOC, colocalizing with the asymmetric VRVs. Concentration of the VRVs at the MTOC was previously observed in ZIKV-infected Huh7 cells (9).

The formation of a vimentin cage-like structure around ubiquitinated protein aggregates located at the MTOC is a specific marker for aggresome formation (21). The formation of a vimentin cage in orthoflavivirus-infected cells was investigated by IFA using an anti-vimentin antibody. Filamentous vimentin was diffusely distributed in the cytoplasm in mock-infected cells as well as in cells infected for 16 h with WNV or ZIKV that contained symmetrically distributed VRVs (Fig. 3I). By 30 hpi, the vimentin had rearranged into a cage-like structure surrounding the asymmetrically concentrated VRVs. Aggregated mitochondria were previously shown to associate with aggresomes (21). Mitochondria detected by IFA using red MitoTracker were broadly distributed in the cytoplasm in uninfected and WNV Eg101-infected cells at 16 hpi (Fig. 3J). However, by 30 hpi, most mitochondria had condensed and colocalized with the asymmetric VRVs. An IFA performed using a Proteostat aggresome detection kit (Enzo) detected a faint, diffuse red signal in mock-infected and WNV-infected cells at 16 hpi. The Proteostat red signal intensity increased and colocalized with the asymmetric VRVs in both WNV-infected and ZIKV-infected A549 cells by 30 hpi (Fig. 3K). Pearson correlation data (Fig. 3L) and fluorescence intensity plots (Fig. 3M) confirmed positive colocalization of the asymmetric VRVs and the Proteostat dye. These data indicate that the asymmetric VRVs observed in both WNV-infected and ZIKV-infected A549 cells have characteristics consistent with those of an aggresome.

### Acetylated, filamentous MTs are required for asymmetric clustering of the VRVs

**Fig 4:**
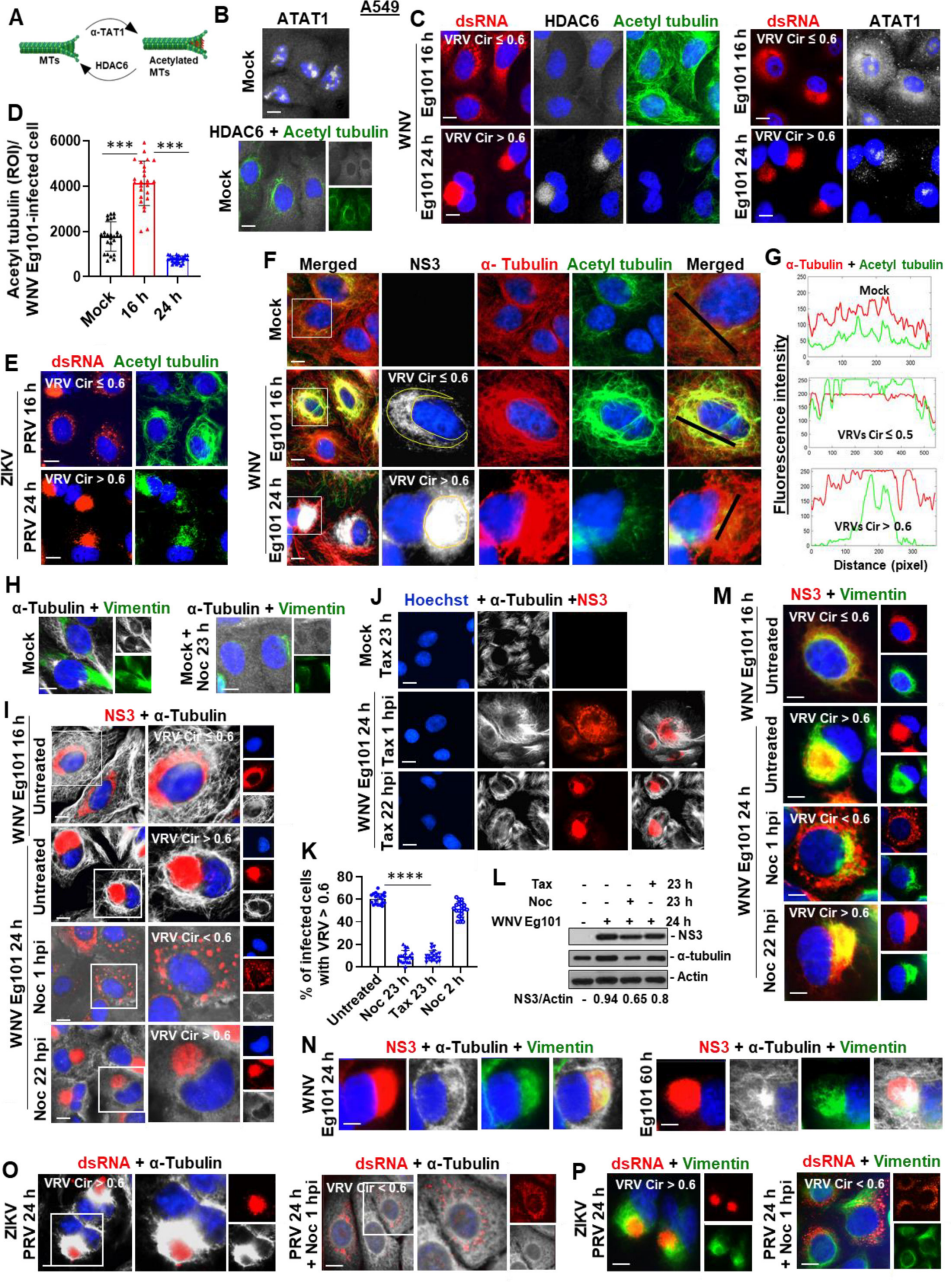
WNV and ZIKV infections induce increased MT acetylation in A549 cells by 16 hpi. (**A**) Diagram of cellular enzymes that acetylate and deacetylate MTs. (**B and C**) Mock- or WNV Eg101-infected (MOI of 3) A549 cells were processed for IFA at 16 and 24 hpi to detect ATAT1 (white), HDAC6 (white), acetyl α-tubulin (green), and VRVs (dsRNA, red). (**D**) Relative signal intensity (ROI) of acetylated MTs per WNV Eg101-infected A549 cell at 16 and 24 hpi. (**E**) ZIKV PRV-infected (MOI of 3) A549 cells at 16 and 24 hpi were processed for IFA to detect VRVs (dsRNA, red) and acetyl tubulin (green). (**F**) Mock- or WNV Eg101-infected (MOI of 3) A549 cells were processed for IFA at 16 and 24 hpi to detect acetyl α-tubulin (green) and α-tubulin (red) in the same cell. VRVs were detected with anti-NS3 antibody (white). A cell with VRVs of Cir ≤ 0.6 at 16 hpi and one with Cir > 0.6 at 24 hpi (indicated by a white box) were enlarged. (**G**) Fluorescence intensity profile plots of the extent of colocalization of α-tubulin and acetylated α-tubulin across the indicated area (black line) in the WNV-infected cells. (**H**) Untreated and 23 h Noc-treated (2 µM) mock-infected A549 cells were processed for IFA to detect α-tubulin (white) and vimentin (green). (**I**) WNV Eg101 (MOI of 3) infected cells were untreated or treated with Noc starting at 1 hpi or 22 hpi and processed for IFA at 16 or 24 hpi to detect NS3 (red) and α-tubulin (white). (**J**) Mock or WNV Eg101 (MOI of 3) infected A549 cells were treated with Tax (1 µM) starting at 1 hpi or 22 hpi and processed for IFA at 24 hpi to detect α-tubulin (white) and NS3 (red). (**K**) Percentage of infected A549 cells with VRVs of Cir > 0.6 at 24 hpi. (**L**) Western blot analysis of NS3 and α-tubulin levels in extracts from untreated, Noc-treated, and Tax-treated WNV Eg101-infected A549 cells at 24 hpi. (**M**) WNV Eg101 (MOI of 3) infected cells were untreated or treated with Noc starting at 1 hpi or 22 hpi and processed for IFA at 16 or 24 hpi to detect NS3 (red) and vimentin (green). (**N**) WNV Eg101-infected A549 cells were processed for IFA at 24 and 60 hpi to detect α-tubulin (white) and vimentin (green) and NS3 (red). (**O**) ZIKV PRV (MOI of 3) infected A549 cells untreated or treated with Noc starting at 1 hpi were processed for IFA at 24 hpi to detect dsRNA (red) and α-tubulin (white). (**P**) ZIKV PRV (MOI of 3) infected A549 cells untreated or treated with Noc starting at 1 hpi were processed for IFA at 24 hpi to detect dsRNA (red) and vimentin (green). Scale bars: 60 µm (µm/pixel [X] and µm/pixel [Y] were adjusted to 1). Statistical significance was determined by Student’s *t*-test. ***P < 0.001 and ****P < 0.0001.

MTs consist of heterodimers of α- and β-tubulin, and their structure and functions are regulated by post-translational modifications (30). α-Tubulin is acetylated by the acetyltransferase ATAT1 at Lys40 on the luminal side of the MTs (31) and is deacetylated by HDAC6 (32) (Fig. 4A). Acetylated tubulin is associated with mechanically stable, polymerized, and filamentous MTs and plays an important role in cell adaptation to stress (33). Deacetylated MTs are susceptible to disassembly or depolymerization, but the dynamic instability provided by deacetylation allows the MTs to grow or shrink (30, 34, 35). ATAT1 changes its intracellular location between the nucleus and the cytoplasm during the cell cycle (36), while HDAC6 is located primarily in the cytoplasm. In mock-infected cells, low-signal-intensity HDAC6 was diffusely distributed in the cytoplasm, ATAT1 was located primarily inside the nucleus, and filamentous acetyl tubulin of low signal intensity was detected in the cytoplasm (Fig. 4B). By 16 hpi with WNV Eg101 (VRVs ≤ 0.6), the acetylated α-tubulin signal intensity had increased, and much of the ATAT1 was diffusely distributed in the cytoplasm, but the distribution and signal intensity of HDAC6 remained similar to that in mock-infected cells (Fig. 4C and D). By 24 hpi, the acetyl α-tubulin signal intensity and cytoplasmic ATAT1 signal intensity had decreased, while HDAC6 had concentrated and colocalized with the asymmetric VRVs. By 16 hpi in ZIKV PRV-infected A549 cells with VRVs of Cir ≤ 0.6, the acetyl α-tubulin signal intensity had increased, and by 24 hpi, it colocalized with asymmetric VRVs (Cir > 0.6) (Fig. 4E). MT dynamics in WNV-infected cells were further investigated with an IFA using both anti-α tubulin and anti-acetyl α-tubulin antibodies. In mock-infected A549 cells, low levels of acetylation of filamentous MTs were detected (Fig. 4F). By 16 hpi with WNV Eg101, α-tubulin acetylation of the filamentous MTs had greatly increased in cells containing symmetrically distributed VRVs (Cir ≤ 0.6). By 24 hpi, the amount of α-tubulin that was acetylated had decreased in infected cells with asymmetric VRVs (Cir > 0.6) and colocalized with the VRVs at the MTOC. The deacetylated MTs were condensed around the asymmetric VRVs and the nucleus. Fluorescence intensity plots of α-tubulin and acetylated α-tubulin across the areas indicated by the black lines in the enlarged merge images confirmed that acetylated α-tubulin had increased by 16 hpi compared to mock-infected cells (Fig. 4G).

To investigate whether polymerized MTs are required for the formation of asymmetric VRVs in infected cells, A549 cells infected with WNV or ZIKV were treated with Nocodazole (Noc), a drug that depolymerizes MTs (37). In mock-infected and WNV Eg101-infected cells at 16 hpi, MTs were filamentous, and the VRVs were symmetrically distributed around the nucleus (Fig. 4H and I 1st row). At 24 hpi, the MTs had condensed to form a cage surrounding the asymmetrically clustered VRVs and the nucleus (Fig. 4I 2nd row and 4K). When the mock-infected cells or cells infected with WNV were treated with Noc starting at 1 hpi for 23 h, the α-tubulin was diffusely distributed throughout the cytoplasm (Fig. 4H), and the VRVs in the WNV-infected cells were symmetrically dispersed in the cytoplasm by 24 hpi (Fig. 4I 3rd row and 4K). When Noc was added to WNV-infected cells at 22 hpi for 2 h, the depolymerized α-tubulin had become diffusely distributed in the cytoplasm, but the VRVs remained asymmetrically clustered at the MTOC (Fig. 4I 4th row and 4K). WNV Eg101-infected A549 cells were also treated with Paclitaxel, also known as Taxol (Tax), a drug that enhances microtubule assembly and promotes the formation of MT bundles in the cells (38). When infected cells were treated with Tax starting at 1 hpi for 23 h, the VRVs were symmetrically dispersed at 24 hpi, but when Tax was added at 22 hpi for 2 h, the VRVs remained asymmetrically clustered (Fig. 4J and K). With both Tax treatments, MT bundles were detected. Reduced viral protein (NS3) levels were observed in both extracts of WNV-infected cells treated with Noc or Tax for 23 h (Fig. 4L). A vimentin cage, which was detected in WNV Eg101-infected cells by 24 hpi, did not form in cells treated with Noc starting at 1 hpi (Fig. 4M). However, an already formed vimentin cage was not affected when Noc was added at 22 hpi. Aggresome formation was previously shown to be inhibited by either Noc or Tax treatment (21, 22, 39). Asymmetrically clustered VRVs surrounded by a vimentin cage were still present in WNV Eg101-infected A549 cells by 60 hpi, but the MT cage was becoming fragmented (Fig. 4N). Addition of Noc to ZIKV PRV-infected cells starting at 1 hpi for 23 h inhibited the formation of asymmetric VRVs surrounded by vimentin and MT cages (Fig. 4O and P). Treatment with Tax was previously shown to inhibit the formation of asymmetric VRVs in ZIKV-infected Huh7 and A549 cells (9, 25).

### Knockdown of HDAC6 prevents asymmetric VRV formation in virus-infected cells

**Fig 5:**
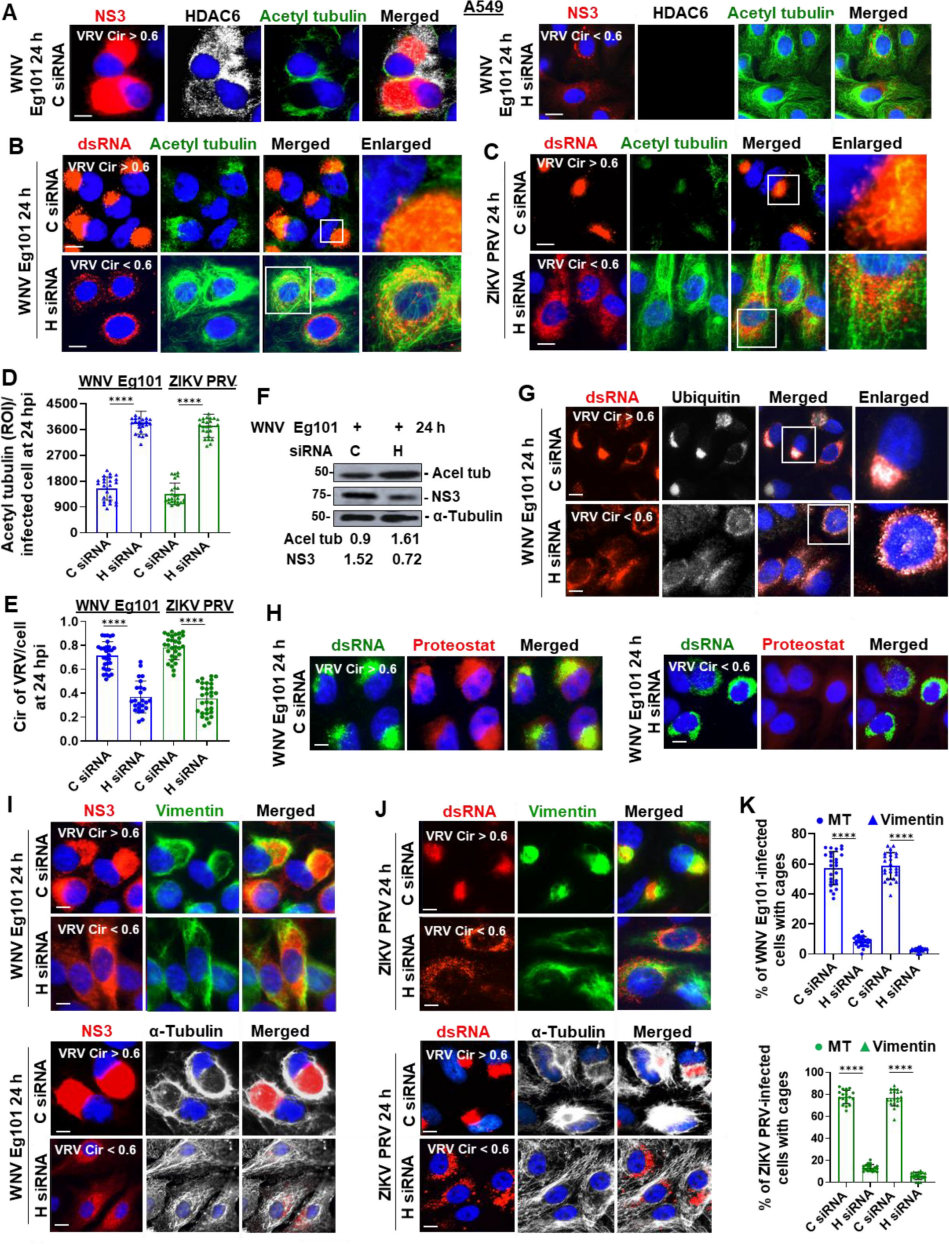
HDAC6 is required for the formation of asymmetric VRVs surrounded by vimentin and MT cages in A549 cells infected with WNV or ZIKV. (**A**) A549 cells were transfected with control (C) or HDAC6 (H) siRNA and infected with WNV Eg101 (MOI of 3) at 24 h after transfection. At 24 hpi, the cells were processed for IFA to detect HDAC6 (white), acetyl-α tubulin (green), and dsRNA (red). (**B**) WNV Eg101 or (**C**) ZIKV PRV (MOI of 3) at 24 h after transfection. At 24 hpi, the cells were processed for IFA to detect acetyl-α tubulin (green) and dsRNA (red). The areas in the white boxes were enlarged. (**D**) Relative intensity (ROI) of acetylated tubulin per infected cell transfected with C or H siRNA at 24 hpi. (**E**) Circularity of the VRV area in cells transfected with C or H siRNA and infected with WNV Eg101 or ZIKV PRV. (**F**) Western blot analysis of cell lysates from WNV Eg101-infected cells transfected with either C or H siRNA 24 h prior to infection. WNV Eg101-infected (MOI 3) A549 cells, transfected with either C or H siRNA 24 h prior to infection, were processed for IFA at 24 hpi to detect (**G**) ubiquitin (white) and dsRNA (red) or (**H**) Proteostat (red) and dsRNA (green). (**I and J**) A549 cells in replicate wells were transfected with either C or H siRNA and infected with WNV Eg101 or ZIKV PRV (MOI of 3) at 24 h after transfection. At 24 hpi, the cells were processed for IFA to detect α-tubulin (white), vimentin (green), and VRVs by NS3 or dsRNA (red). (**K**) Quantification of the percentage of WNV Eg101-infected or ZIKV PRV-infected cells with MT cages (circles) and vimentin cages (triangles). Scale bars, 60 µm (µm/pixel [X] and µm/pixel [Y] were adjusted to 1). Statistical significance was determined by a Student’s *t*-test. ****P < 0.0001.

Whether HDAC6 function was required for the formation of asymmetric VRVs was next investigated. A549 cells were transfected with either control siRNA or HDAC6 siRNA 24 h prior to infection with WNV Eg101 or ZIKV PRV, and the cells were processed for IFA at 24 hpi. In infected cells transfected with control (C) siRNA, the VRVs were asymmetrically clustered and colocalized with concentrated HDAC6 and a small amount of acetylated MTs at the MTOC (Fig. 5A through D). In contrast, highly acetylated and filamentous MTs, no detectable HDAC6 and symmetrically distributed VRVs were detected in infected cells transfected with HDAC6 (H) siRNA. The Cir value of the VRVs remained low in both WNV- and ZIKV-infected cells transfected with HDAC6 siRNA (Fig. 5E). A western blot analysis of WNV Eg101-infected cell lysates confirmed increased levels of acetylated α-tubulin in infected cells transfected with HDAC6 siRNA (Fig. 5F). Viral protein levels were reduced in cells transfected with HDAC6 siRNA. The anti-HDAC6 antibody detected HDAC6 by IFA but not by western blotting.

In WNV-infected cells transfected with control siRNA, ubiquitinated proteins and Proteostat were concentrated and colocalized with asymmetrically localized VRVs (Fig. 5G and H). In contrast, both ubiquitinated proteins and VRVs were symmetrically dispersed in the cytoplasm, and Proteostat staining was not detected by 24 hpi in WNV-infected cells transfected with HDAC6 siRNA, indicating that HDAC6 was required to form aggresome-like asymmetric VRVs. While both vimentin and MT cage-like structures surrounding the asymmetric VRVs were detected in infected cells transfected with control siRNA, neither a vimentin nor an MT cage formed in infected cells transfected with HDAC6 siRNA (Fig. 5I and J). HDAC6 knockdown reduced the percentage of WNV-infected cells containing MT and vimentin cages from ~60% to ~7% (Fig. 5K) and from ~80% to ~10% in ZIKV-infected cells.

**Fig 6:**
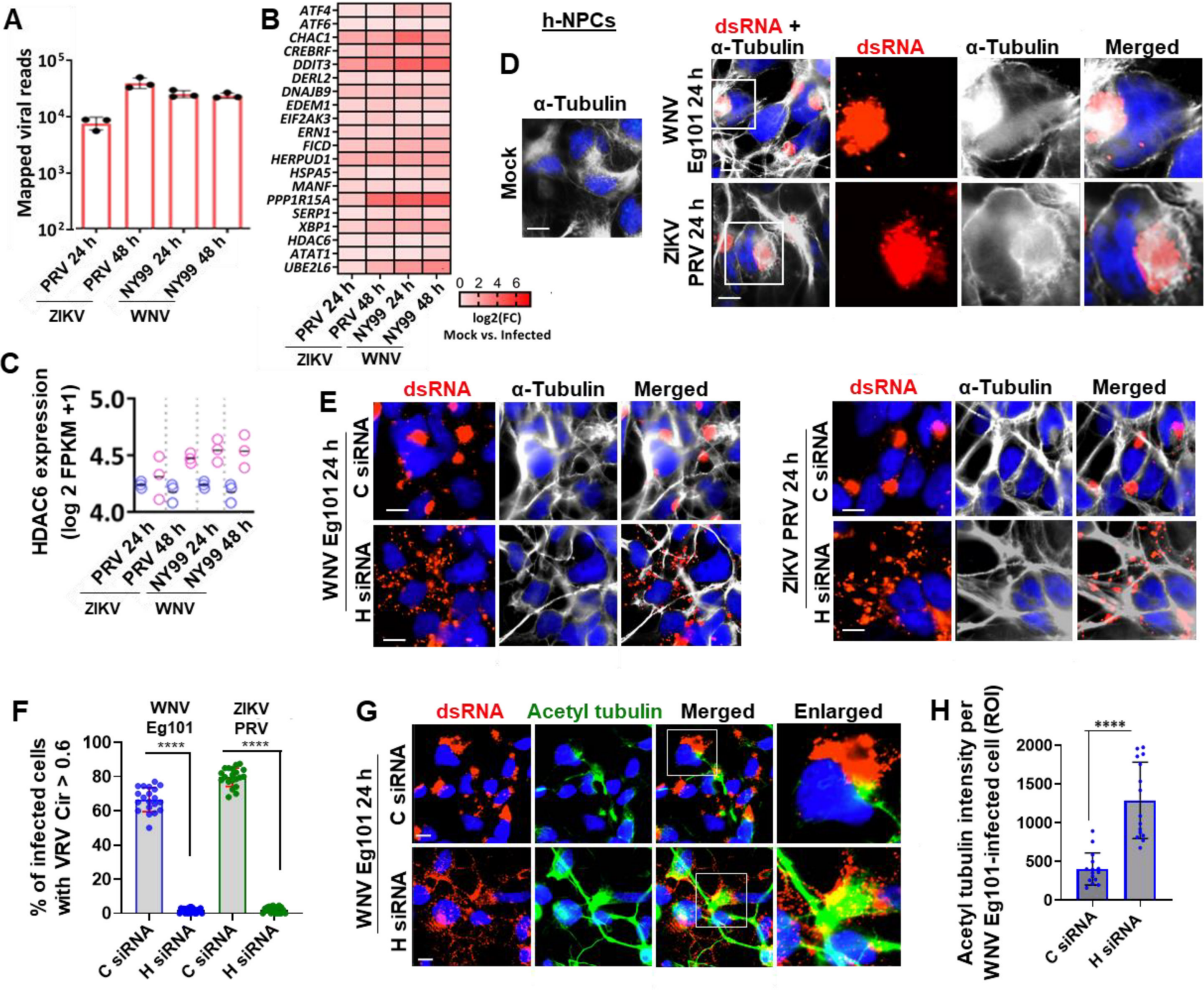
HDAC6 is required for the formation of asymmetric VRVs surrounded by MT cages in h-NPCs infected with WNV or ZIKV. (**A**) Relative intracellular viral RNA levels in h-NPCs infected with WNV NY99 or ZIKV PRV (MOI of 1) for 16 or 32 h determined by RNAseq analysis. (**B**) UPR pathway genes upregulated by the WNV and ZIKV infections. A gene ontology analysis of the RNA-seq data. (**C**) *HDAC6* mRNA expression in h-NPCs infected with WNV NY99 or ZIKV PRV by 24 and 48 hpi. (**D**) h-NPCs infected with WNV Eg101 or ZIKV PRV (MOI 3) were processed for IFA at 24 hpi to detect α-tubulin (white) and VRVs (dsRNA, red). The areas in the white boxes were enlarged. (**E**) h-NPCs were transfected with control (C) or HDAC6 (H) siRNA 24 h prior to infection with WNV Eg101 or ZIKV PRV (MOI 3). At 24 hpi, the cells were processed for IFA to detect α-tubulin (white) and dsRNA (red). (**F**) Percentage of infected cells with VRVs of Cir ≥ 0.6. (**G**) h-NPCs were transfected with C or H siRNA, infected with WNV Eg101 (MOI of 3) 24 h after transfection, and processed for IFA to detect acetyl tubulin (green) and dsRNA (red) at 24 hpi. (**H**) Quantification of acetyl tubulin intensity (ROI)/cell. Scale bars: 60 µm (µm/pixel [X] and µm/pixel [Y] were adjusted to 1). Statistical significance was determined by a Student’s *t*-test. ****P < 0.0001.

WNV Eg101 and ZIKV PRV infections efficiently induced the formation of asymmetric VRVs in h-NPCs (Fig. 1K and L). UPR activation by these infections was next investigated in h-NPCs. Total cellular RNA was extracted from h-NPCs at 24 and 48 hpi with WNV NY99 or ZIKV PRV, polyA-selected prior to library construction, and subjected to RNA-seq analysis. The WNV NY99 infection produced a higher level of intracellular viral RNA than the ZIKV infection by 24 hpi, but viral RNA levels were higher in the ZIKV-infected h-NPCs by 48 hpi (Fig. 6A; Table S5). Transcriptomic and gene ontology analyses of the RNA-seq data followed by a gene ontology analysis showed that both the WNV and ZIKV infections in h-NPCs upregulated multiple UPR pathway genes (Fig. 6B; Table S6). *HDAC6* mRNA expression was upregulated by both the WNV NY99 and ZIKV PRV infections in three replicate samples for each infection by 24 hpi (Fig. 6C; Table S7). Asymmetric VRVs were detected in ~70% of WNV Eg101-infected and ~80% of ZIKV PRV-infected h-NPCs (Fig. 1J and K). An MT cage-like structure was detected around asymmetric VRVs and the nucleus in h-NPCs infected with WNV or ZIKV by 24 hpi (Fig. 6D). Transfection of WNV- or ZIKV-infected h-NPCs with HDAC6 siRNA prior to infection reduced the percentage of cells with an MT cage and asymmetrically clustered VRVs from ~70% to ~5% and from ~80% to ~5%, respectively, at 24 hpi (Fig. 6E and F). Transfection of WNV Eg101-infected h-NPCs with HDAC6 siRNA prior to infection increased the acetyl tubulin signal intensity by ~3-fold by 24 hpi and inhibited asymmetric clustering of VRVs (Fig. 6G and H). These data suggest that HDAC6 expression is required for the formation of asymmetric WNV and ZIKV VRVs in both A549 cells and h-NPCs.

### Asymmetric VRV formation in WNV- and ZIKV-infected cells delays activation of cell death pathways and increases the viral yield

**Fig 7:**
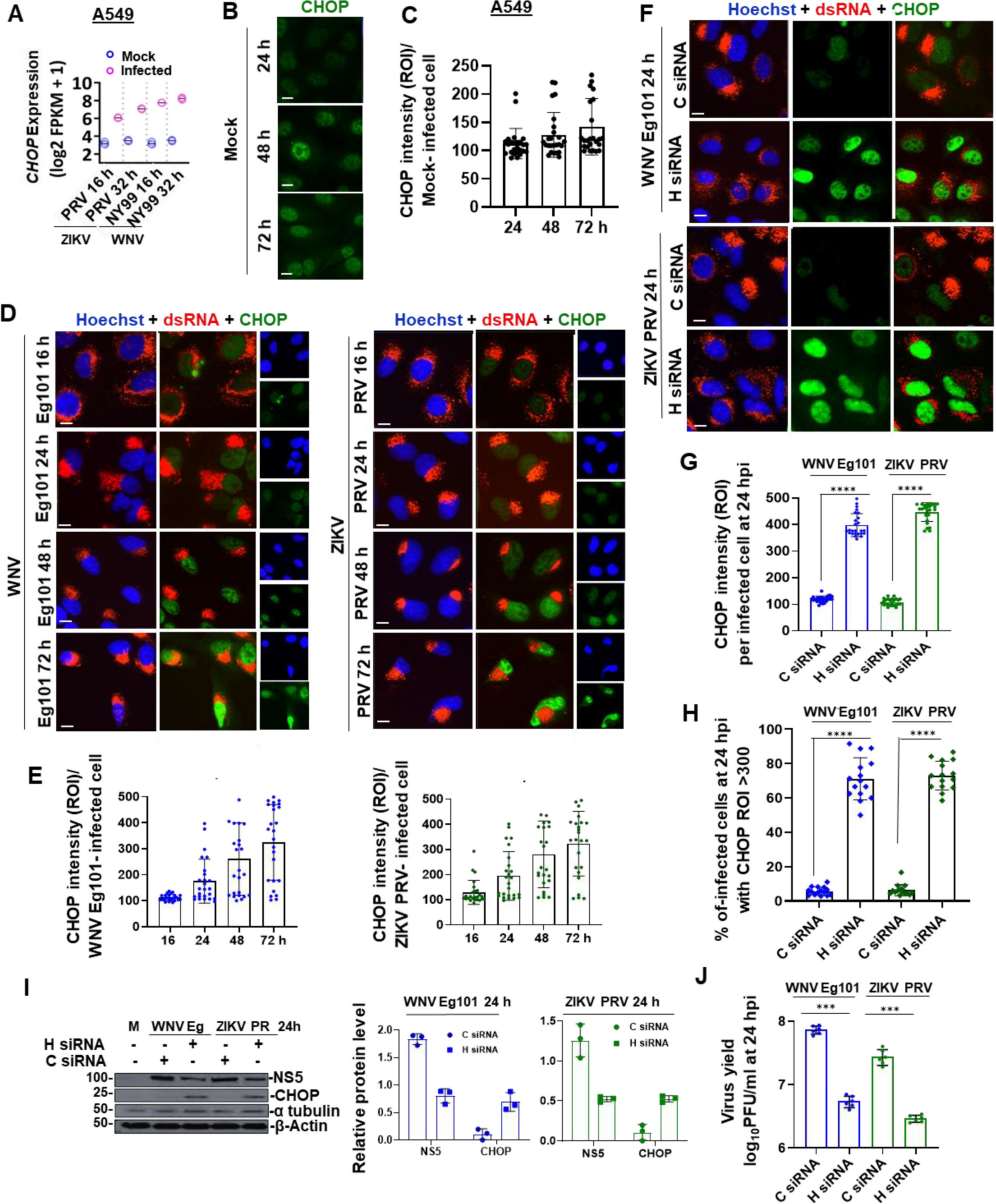
Asymmetric VRV formation in WNV- and ZIKV-infected cells delays apoptosis and increases the viral yield. (**A**) Expression of CHOP at mRNA level in both WNV NY99 and ZIKV PRV- infected A549 cells by 16 and 32 hpi. (**B**) Detection of CHOP protein (green) by IFA in mock-infected A549 cells at indicated times. (**C**) Quantification of nuclear CHOP protein intensity (ROI)/cell. (**D**) WNV Eg101- or ZIKV PRV-infected (MOI 3) A549 cells were processed for IFA to detect CHOP (green) with anti-CHOP and VRVs (red) with anti-dsRNA antibodies at the indicated time. (**E**) Quantification of nuclear CHOP intensity (ROI) per WNV Eg101- and ZIKV PRV-infected cells at different times after infection. (**F**) A549 cells infected with either WNV Eg101 or ZIKV PRV (MOI 3) were either transfected with C siRNA or H siRNA 24 h prior to infection. At 24 hpi, cells were processed for IFA with antibodies to detect CHOP (green) and VRVs (red). (**G**) Quantification of nuclear CHOP intensity (ROI) per WNV Eg101- and ZIKV PRV- infected cells. (**H**) Quantification of virus-infected and CHOP-expressing cells with nuclear ROI > 300. (**I**) A549 cells infected with either WNV Eg101 or ZIKV PRV (MOI 1) were transfected with either control (C) siRNA or HDAC6 (H) siRNA 24 h prior to infection, and the cell lysates at 24 hpi were processed for western blot analysis. Protein band intensity was quantified using ImageJ and normalized against β-actin. (**J**) A549 cells infected with either WNV Eg101 or ZIKV PRV (MOI 1) and transfected with either C siRNA or H siRNA 24 h prior to infection. Media harvested at 24 hpi were used to assay viral titer by plaque assay. Scale bars: 60 µm (µm/pixel [X] and µm/pixel [Y] were adjusted to 1). Statistical significance was determined by Student’s *t*-test. ***P < 0.001 and ****P < 0.0001.

The role of the UPR is to prevent cell damage and restore a homeostatic state. However, if the UPR cannot alleviate excessive or prolonged ER stress, the UPR switches from a pro-survival to a pro-apoptotic response (17). A component of the pro-apoptotic response is efficient translation of the second ORF of the *DDIT3* (*CHOP*) mRNA. CHOP protein translocates to the nucleus, where it activates the expression of cell death pathway genes (20). *CHOP* mRNA levels were rapidly and robustly upregulated by WNV and ZIKV infections in A549 cells and h-NPCs (Fig. 2B and 6B; Fig. 7A). An IFA indicated that the CHOP protein signal intensity in the nucleus was faint (average ROI <150) in mock-infected A549 cells (Fig. 7B and C). Analysis of CHOP signal intensity in A549 cells infected with WNV Eg101 or ZIKV PRV over time indicated that most infected cells had a low average CHOP signal intensity in the nucleus (ROI ≤ 100–150) at 16 and 24 hpi, with 5–7% of the infected cells with a CHOP signal intensity (ROI > 300) at 24 hpi (Fig. 7D and E). The percentage of infected cells with a CHOP signal intensity >300 (indicative of CHOP death pathway activation) increased as the infection progressed, and at 72 hpi, even though almost all the infected cells contained asymmetrically clustered VRVs, CHOP levels were high (ROI ≥ 300) in ~50% of the infected cells. In contrast, ~75–80% of A549 cells transfected with HDAC6 siRNA and then infected with WNV Eg101 or ZIKV PRV 24 h later contained very high CHOP nuclear signal intensity by 24 hpi (Fig. 7F through H). By 24 hpi, infected cells transfected with control (C) siRNA contained asymmetric VRVs and did not contain increased CHOP levels. CHOP protein was not detected in a western blot analysis of lysates from A549 cells transfected with control (C) siRNA for 24 h and then infected with WNV Eg101 or ZIKV PRV for 24 h (Fig. 7I). In contrast, CHOP protein was detected in A549 cell lysates transfected with HDAC6 (H) siRNA 24 h prior to infection with WNV or ZIKV. In HDAC6 siRNA-transfected, infected cells, CHOP protein levels increased by ~70%, whereas viral NS5 protein levels decreased by ~35–40%. Virus titers in media harvested at 24 hpi from A549 cells transfected with either control siRNA or HDAC6 siRNA 24 h prior to infection with WNV Eg101 or ZIKV PRV (MOI 1) were determined by plaque assay (Fig. 7J). A decrease of approximately 1 log in the virus infectivity titer was observed for the WNV- and ZIKV-infected cells transfected with HDAC6 siRNA compared to those transfected with the control siRNA.

## DISCUSSION

The formation of a single large cluster of VRVs at the MTOC, surrounded by intermediate filaments and MTs that had reorganized to form a cage-like structure, was initially detected by IFA in Huh7 cells and h-NPCs infected with ZIKV MR766 or ZIKV H/PF/2013 (9). Asymmetric VRVs were subsequently detected in A549 cells infected with ZIKV PRV but not in A549 cells infected with WNV Kun (25). We did not observe asymmetrically clustered VRVs in our previous studies of WNV infections in BHK21 cells but did detect them in subsequent studies of WNV Eg101 and ZIKV PRV infections in A549 cells and h-NPCs. We confirmed that a WNV Kun infection did not induce asymmetrically clustered VRVs in A549 cells. These data suggested virus strain-specific and cell type-specific variation in the induction of asymmetrically clustered orthoflavivirus VRVs. To determine the molecular mechanisms involved, the sequential events in A549 cells infected with WNV or ZIKV that result in VRV clustering at the MTOC were investigated.

Protein aggregates do not accumulate in unstressed cells due to the functioning of the cellular quality control machinery, which maintains homeostasis by regulating the fidelity of transcription and translation, chaperoning nascent and unfolded proteins, and selectively degrading misfolded polypeptides before they aggregate (24). If not adequately chaperoned, misfolded integral membrane proteins dislocate from the ER membrane, unfold, become ubiquitinated, and are degraded by the proteasome. Alternatively, the hydrophobic regions of membrane proteins facilitate their aggregation, and the aggregates are transported to an aggresome (21). An aggresome is a single, membrane-less, cytoplasmic aggregate of ubiquitinated, unfolded and/or misfolded proteins that is located at the MTOC and enclosed in a “cage” of the intermediate filament vimentin (21, 24). Aggresome formation was initially discovered in cells overexpressing recombinant proteins, such as cystic fibrosis transmembrane regulator protein or presenilin protein in HEK293 cells (21) or a GFP chimera in COS-7 cells (22). Overexpressed proteins with multiple hydrophobic regions and ubiquitination sites have a greater propensity for inducing aggresome formation. However, triggering aggresome formation by overexpression of the same recombinant protein varied with cell type (40). Aggresome formation is initiated when the amount of ubiquitinated, unfolded, and/or misfolded protein that has aggregated in the ER lumen exceeds the cell-specific threshold that can be managed by the UPR. Cytoplasmic HDAC6 levels increase, and the accumulated ubiquitinated protein aggregates interact with HDAC6 through its ubiquitin-binding domain. The HDAC6 dynein motor binding domain interacts with acetylated polymerized MTs and delivers the protein aggregates to the cellular perinuclear region near the MTOC by dynein-dependent retrograde transport (23). Vimentin forms a cage-like structure surrounding the trafficked aggregated proteins (21, 24). Although acetylated polymerized MTs are required for aggresome formation, deacetylation and depolymerization of MTs by treatment of cells with Noc after an aggresome has formed do not disperse the aggresome, indicating that it is not a dynamic structure (22). Mitochondria and heat shock chaperone proteins have been observed to concentrate around an aggresome.

Orthoflavivirus genomes encode a single long polyprotein with many hydrophobic transmembrane regions. The mature E, preM, and NS1 proteins, which have transmembrane domains, are located on the lumen side of the ER membrane. Eight of the 10 mature orthoflaviviral proteins have been shown to become ubiquitinated in infected cells (41). These characteristics would enhance ubiquitinated aggregate formation. Orthoflavivirus proteins are intimately associated with and extensively remodel the ER of infected cells by inducing convoluted ER membranes used for translation of the long viral polyprotein, ER membrane invaginations (VRVs) used for viral genome RNA synthesis, and ER associated with viral envelope and capsid proteins used for nascent virion assembly. VRVs are initially symmetrically distributed in the ER surrounding the nucleus and increase in number during the first 16 h of the virus replication cycle in A549 cells. Acetylation of filamentous tubulin increases, facilitating the extensive remodeling and expansion of the ER required to accommodate escalating orthoflavivirus protein translation, genome RNA replication, and virion assembly (42). Orthoflavivirus infections have previously been shown to induce ER stress and to activate the UPR (10–12). Our transcriptomic and western blot data confirmed that WNV NY99 and ZIKV PRV infections in both A549 cells and h-NPCs induced upregulation of multiple UPR genes and activated downstream UPR pathways. Cytoplasmic levels of ubiquitinated proteins and HDAC6 increased by 24 hpi, and viral structural and nonstructural proteins, dsRNA (VRVs), HDAC6, GRP78, ubiquitin, and calnexin were observed to concentrate and colocalize in a single asymmetric cluster near the MTOC, surrounded by a vimentin cage. Knockdown of *HDAC6* mRNA or disruption of the acetylated MT network by Noc or Tax treatment of infected cells at 1 hpi prevented the formation of asymmetrically clustered VRVs, consistent with HDAC6 and MTs being required for trafficking ubiquitinated, aggregated viral proteins and VRVs, along with associated ER to the MTOC. Vimentin and MTs subsequently reorganize to form cage-like structures around the VRVs clustered at the MTOC. The sequential steps required for the formation of asymmetrically clustered ZIKV and WNV VRVs were found to be the same as those previously shown to be required for aggresome formation. Neither an MT cage surrounding an aggresome induced by overexpression of a recombinant cellular protein nor the presence of ER in these aggresomes has been reported. The detection of viral structural and nonstructural proteins, viral dsRNA, and the ER marker calnexin concentrated in the aggresomes strongly suggests that ER membrane is in an aggresome formed in an orthoflavivirus-infected cell. The accumulation of remodeled ER was also observed in ZIKV-infected h-NPCs by Cortese et al. (9).

The efficiency of induction of aggresome formation has been observed to vary with the infecting orthoflavivirus and cell type. In the present study, the ZIKV PRV infection induced aggresome formation in A549 cells more efficiently than the WNV infections. ZIKV NS1 has extended hydrophobic regions and a unique hydrophobic spike in its wing domain compared to the NS1s of other orthoflaviviruses (43, 44). Also, the first two predicted transmembrane domains of ZIKV NS4B are more hydrophobic than those of other orthoflaviviruses (45). These extended hydrophobic regions would be expected to increase aggregation (46). Although WNV Eg101, WNV NY99, and WNV B956 infections efficiently induced aggresome formation in A549 cells, neither the WNV Kun nor WNV W956IC infections did. The inability of these two infections to induce aggresome formation was due to the lower amounts of viral proteins produced, which did not reach the threshold for triggering the sequential stress responses required for aggresome formation. WNV Kun replicates inefficiently in A549 cells, producing lower levels of viral protein and virus yields than a WNV NY99 infection due to the enhanced sensitivity to the cellular type 1 interferon response (27). The delayed amplification of W956IC proteins enhances the sensitivity of this virus to the type 1 interferon response. Asymmetric VRVs were not detected by IFA using anti-dsRNA antibody in our previous studies of BHK21 cells or mouse embryofibroblast cell lines infected with WNV Eg101, even though viral replication was efficient in these cells (26, 47, 48). Cell type-specific variation is likely due to differences in the thresholds of unfolded/misfolded proteins required to trigger the UPR and subsequent aggresome response and in the efficiency of activation of the cellular chaperone system.

Although *DDIT3* (CHOP) mRNA is rapidly upregulated when the UPR is activated, only the uORF in the 5′ leader of this mRNA is translated while ER homeostasis is maintained (19). When ER homeostasis becomes disrupted, p-eIF2α facilitates ribosomal bypass of the uORF due to its poor start codon context, resulting in translation initiation at the downstream CHOP ORF. Our RNAseq data indicated that both ZIKV and WNV infections efficiently upregulate *DDIT3* (CHOP) mRNA by 16 hpi in A549 cells, but CHOP protein is not detected in the nucleus until 48 hpi, with the levels increasing by 72 hpi. In contrast, high nuclear levels of CHOP protein were detected by 24 hpi in A549 cells when HDAC6 expression was knocked down, indicating that these cells were unable to maintain ER homeostasis and had more rapidly transitioned from a stress alleviation program to a programmed cell death program (49). HDAC6 knockdown in WNV-infected A549 cells and h-NPCs inhibited aggresome formation and also reduced viral protein levels and virus yields. Aggresome formation delayed activation of the death pathways and extended the time of virus production. The asymmetric VRVs persisted in A549 cells through 72 hpi. These cells continued to efficiently produce intracellular viral proteins and dsRNA, assemble nascent virions within ER membrane surrounded by vimentin and MT cages, and secrete virions into the extracellular space.

Our data indicate that efficient viral protein production during both WNV and ZIKV infections in A549 cells and h-NPCs results in aggresome formation, which is an additional “backup” cellular stress response. Although this response is not required for efficient orthoflavivirus replication, it does have proviral effects. The “compaction” of modified ER that is associated with viral protein translation, viral RNA replication, and virion assembly in an aggresome would be expected to increase the efficiency of virion production. The vimentin and MT cages surrounding an aggresome might reduce IFN pathway-mediated restriction of viral replication due to the exclusion of ISG mRNAs and proteins. An aggresome apparently does not prevent nascent virus from exiting an infected cell since these cells continued to produce extracellular virions. h-NPCs are the main target cells of ZIKV infections in the developing fetal brain (9). h-NPCs can undergo symmetric or asymmetric divisions, producing new progenitor cells and/or immature neurons (16). ZIKV infections have been shown to dysregulate the expression of h-NPC genes involved in neurogenesis, inflammation, cell cycle regulation, and cell death pathways, altering normal hNPC proliferation and differentiation, and potentially leading to impaired brain expansion (5, 50–53). Delayed activation of death pathways in ZIKV-infected h-NPCs due to aggresome formation could increase viral spread in the brain and could alter the differentiation and function of the daughter cells generated (54).

## MATERIALS AND METHODS

### Cells

A549, adenocarcinomic human alveolar basal epithelial cells (CCL-185), were purchased from ATCC and maintained in DMEM/F-12K medium (Gibco #11330032) supplemented with 10% fetal bovine serum (FBS) (R & D Systems, #11150), 1% Pen/Strep (Gibco, #15140-122), and 1% L-glutamine (Gibco, #25030-081). BHK21 cells were obtained from T. Wiktor, Wistar Institute, and Vero cells were obtained from ATCC. Both BHK21 and Vero cells were maintained in MEM (no glutamine) (Gibco, #11090081) supplemented with 7.5% FCS, 1% Pen/Strep, 1% sodium pyruvate (Gibco, #11360-070), 1% L-glutamine (Gibco, #25030-081), and 1% NEAA (Gibco, #11140-050).

Human-induced pluripotent stem cell colonies detached from the feeder layer with 1 mg/mL of collagenase type IV treatment (Gibco, #17104-019) were suspended in embryoid body (EB) media, consisting of DMEM/F12 (Life Tech, #11330057) supplemented with 20% KOSR (Life Tech, # 310828028), 2 mM Glutamax (Life Tech, #35050079), 0.1 mM NEAA (Life Tech, #11140076), 0.1 mM 2-ME (Life Tech #21985023), 2 µM dorsomorphin (Tocris Bio, #3093), and 2 µM A-83 (Tocris Bio, #2939), in non-treated polystyrene plates for 4 days with a daily media change. On day 6, the floating EBs were then transferred to Matrigel-coated 6-well plates at day 7 to form neural tube-like rosettes. EB media was replaced by neural induction media (h-NPC media) consisting of 1:1 DMEM/F12 (Life Tech, #11330057) and neurobasal medium (Life Tech, #21103049), 2 mM Glutamax (Life Tech, #35050079), 0.1 mM NEAA (Life Tech, #11140076), B27 (Life Tech, #17504044), N2 supplement (Life Tech, #17502-048), and 1 µM cyclopamine (CellagenTech, #C2925-50). The attached rosettes were kept for 16 days with an h-NPC media change every other day. On day 22, the rosettes were picked mechanically and transferred to low attachment plates in hNPC media. On day 24, hNPC aggregates were observed to form spherical structures. hNPC spheres were dissociated into single cells using collagenase IV, followed by seeding onto Matrigel-coated plates.

### Viruses

Viruses used in the studies are WNV Eg101, WNV NY99, WNV B956, ZIKV PRVABC59 (PRV), and W956IC chimera virus. A stock of WNV, strain Eg101 (obtained from J.S. Porterfield, National Institute for Medical Research, Mill Hill, London, UK) was prepared by infecting confluent monolayers of BHK 21 cells at a multiplicity of infection (MOI of 0.1) and harvesting culture fluid at 32 hpi. Clarified culture fluid was aliquoted and stored at −80°C. Stocks of WNV NY99 and WNV B956 (obtained from R. Tesh, University of Texas Medical Branch, World Reference Center for Emerging Viruses and Arboviruses, Galveston, Texas) and WNV Kunjin MRM 16 (BEI NR 51,653) were grown in BHK21 cells as described above for WNV Eg101. An aliquot of the W956IC chimera virus was prepared from the full-length infectious clone in pBR322 that was provided by V. Yamshchikov (55). W956IC virus stocks were prepared by transfecting BHK21 cells with *in vitro*-transcribed full-length genomic RNA as described previously (56). Stock pool of ZIKV PRVABC59 (BEI NR-50240) (PRV) was prepared by infecting ~80% confluent Vero cell cultures at a MOI of 0.1. For ZIKV, the culture media was replaced with fresh media at 24 hpi and harvested at 48 hpi. The infectivity titers of stocks were determined by plaque assay on BHK21 cells for WNV and on Vero cells for ZIKV. Virus samples were serially diluted 10-fold and incubated on cell monolayers for 1 h at 37°C. The virus inoculum was then removed and overlay medium was added. For WNV, the overlay was a 1:1 (vol/vol) mixture of 1% Seakem ME agarose (Lonza #50014) in water and 2× MEM (Gibco, #11430-030) that contained 2.5% FCS, 2.1 µg/µL NaHCO3, and 20 µg/mL of gentamicin. At 72 hpi, the agarose plugs were removed, and the plaques visualized by staining the cells with 0.05% crystal violet in 10% ethanol. For ZIKV plaque assays, monolayers were overlaid with a 1:1 mixture of 1.2% microcrystalline cellulose (Sigma, #435244) in water and 2× MEM (Gibco, #11430-030). The cells were fixed with 4% paraformaldehyde and stained with 1% crystal violet to visualize the plaques. The virus stock titers were: WNV Eg101 (4.3 × 10^7^ PFU/mL), WNV NY99 (3.7 × 10^7^ PFU/mL), WNV Kunjin (2 × 10^6^ PFU/mL), WNV W956IC (5 × 10^7^ PFU/mL), and ZIKV PRV (4.1 × 10^7^ PFU/mL). During experimental studies, A549 cells or h-NPCs were infected with any of these pre-titrated viruses at MOI as indicated in specific figure legend, and viral infectivity was determined by immunostaining or by determining virus titer.

### Indirect immunofluorescence assay (IFA)

Mock-infected or virus-infected cells on coverslips (Fisher brand #12541000) in 24-well plates were fixed at different times after infection with 4% paraformaldehyde in phosphate-buffered saline (PBS) (Sigma #P3813) for 10 min at room temperature, followed by permeabilization with cold 0.1% Triton X-100 in PBS (12 min), and washing with 1× PBS and then incubation in blocking buffer consisting of 5% horse serum (Gibco, #16050-130) in 1× PBS for 1 h at room temperature. Cells were incubated with the following primary antibodies: mouse anti-dsRNA J2 (Jena Biosciences #RNT-SCI-10010200, 1:1000); goat anti-WNV NS3 (R&D Systems, #AF2907, 1:400); mouse anti-ZIKV NS5 (Biofront Technology, #BF-8B8, 1:400); rabbit anti-GRP78/BiP (Proteintech, #11587-1-AP, 1:400); rabbit anti-p-eIF2α S51 (Invitrogen, #PA5-118533, 1:200); goat anti-GADD34 (Abcam #ab9869, 1:200); rabbit anti-G3BP1 (Proteintech, #13057-2-AP, 1:400); mouse anti-α-tubulin (Cell Signaling Technology, #3873, 1:400); rabbit anti-α-tubulin (11H10) (Cell Signaling Technology, #2125, 1:400); rabbit anti-acetyl-α-tubulin (Lys40) (Cell Signaling Technology, #5335, 1:400); mouse anti-acetyl-α-tubulin (Lys40), (Proteintech, #66200-1-Ig, 1:400); rabbit anti-gamma tubulin (Proteintech, #5176-1-AP, 1:200); rabbit anti-vimentin (Cell Signaling Technology, #5741, 1:400); rabbit anti-ubiquitin (Cell Signaling Technology, #20326, 1:300); rabbit anti-HDAC6 (Cell Signaling, #7558, 1:300); rabbit anti-calnexin (Abcam #ab22595, 1:300); rabbit anti-CHOP (Proteintech, #15204-1-AP, 1:300); and mouse anti-nestin clone 10C2 human-specific (Millipore Sigma, #MAB5326, 1:300). Primary antibodies were diluted in blocking buffer overnight at 4°C, washed three times for 10 min with 1× PBS, and then incubated with an Alexa Fluor-conjugated secondary antibody (1:400) (donkey Alexa Fluor 488 anti-rabbit, Invitrogen, #A21206; donkey Alexa Fluor 488 anti-mouse, Invitrogen, #A21202; donkey Alexa Fluor 488 anti-goat, Invitrogen, #A11055; donkey Alexa Fluor 555 anti-rabbit, Invitrogen, #A31572; donkey Alexa Fluor 555 anti-mouse, Invitrogen #A31570; donkey Alexa Fluor 594 anti-goat, Invitrogen, #A11058; donkey Alexa Fluor 647 anti-rabbit, Invitrogen #A31573; donkey Alexa Fluor 488 anti-mouse, Invitrogen, #A31571; donkey Alexa Fluor 488 anti-goat, Invitrogen, #A21447) and 0.5 μg/mL of Hoechst 33258 dye (Invitrogen, #H3570) in blocking buffer for 1.5 h. The coverslips were washed extensively in 1× PBS and mounted on slides with Prolong Gold antifade mounting media (Invitrogen, #P36930). Images were acquired with an Axio Observer Z1 microscope (Zeiss) using a 63× oil immersion objective and processed with Volocity software. For higher resolution images, stacks were recorded at 200 nm intervals and subjected to “iterative deconvolution” using Volocity acquisition software (Perkin Elmer).

### Western blotting

Cells grown to ~95% confluency in 6-well plates were mock-infected or infected with a virus. At various times after infection, cells were washed with PBS and lysed by addition of 150 μL of cell lysis RIPA buffer (25 mM Tris, pH 7.5, 150 mM NaCl, 1% NP-40, 0.1% SDS, 0.5% sodium deoxycholate, Halt protease, and phosphatase inhibitor single use cocktail [Thermo Scientific, #78430]). After separation by SDS-PAGE, proteins were transferred to a 0.45 μM nitrocellulose membrane (Bio-Rad, #88018). The membrane was blocked with blocking buffer made of 5% milk in TBST (10 mM Tris-chloride, 150 mM NaCl, and 0.5% Tween 20) or 0.5% BSA in TBST (for anti-phospho antibodies), followed by incubation with primary antibodies overnight at 4°C, washed three times for 10 min with TBST, and then incubated with secondary antibody in blocking buffer for 1.5 h. Primary antibodies were goat anti-WNV NS3 (R&D Systems, #AF2907, 1: 1000); mouse anti-ZIKV NS5 (Biofront Technology, #BF-8B8, 1:1,000); mouse anti-β-actin antibody (Abcam, #ab8226, 1: 10,000); rabbit anti-PERK (Cell Signaling Technology, #3192, 1:1,000); rabbit p-PERK (Cell Signaling Technology, #3179, 1:500); rabbit anti-p-eIF2α S51 (Invitrogen, #PA5-118533, 1:500); rabbit anti-acetyl-α-tubulin (Lys40) (Cell Signaling Technology, #5335, 1:500); rabbit anti-GRP78/BiP (Proteintech, #11587-1-AP, 1:1,000); and rabbit anti-CHOP (Proteintech, #15204-1-AP, 1: 500). Secondary antibodies were anti-rabbit HRP (Cell Signaling Technology, #7074, 1:1,000), anti-mouse HRP (Cell Signaling Technology, #7076, 1:1,000), and anti-goat HRP (Abcam, #6885, 1:5,000). Western blots were developed using SuperSignal West Pico chemiluminescent substrate (Thermo Scientific, #32106), and the signal was detected by autoradiography. The band intensities of selected proteins were analyzed using ImageJ software, and the values were compared to the band intensity of actin.

### Chemicals

Nocodazole (Noc) (Santa Cruz Biotechnology #CAS 31430-18-9) and Paclitaxel (Taxol, Tax) (Sigma #PHL89806) solutions were made in DMSO. Mock or infected cells in 24-well plates were treated with 2 µM Noc or 1 µM Taxol at the indicated time in the figure. The final concentration of DMSO in culture media for reagents dissolved in DMSO was less than 1%. MitoTracker Red-CMXRos (Invitrogen, #M7512) was added to the media in mock or virus-infected A549 cells before fixing at a final concentration of 500 nM to study mitochondrial dynamics. After a 5-minute incubation, cells and media were removed, and then cells were washed with 1× PBS and fixed for IFA. Proteostat aggresome detection kit (Enzo, #ENZ 51,035) was used to detect aggresome by IFA.

### siRNA transfection

Cells seeded in a 6-well or a 24-well plate at ~70% confluency were transfected with either *HDAC6* siRNA (ThermoFisher Scientific #AM16708) or control siRNA (Santa Cruz Biotechnology #sc-37007) 24 h before infection. Lyophilized siRNA was resuspended in RNAse-free water to make a 10 µM solution. *HDAC6* siRNA or control siRNA was diluted in Opti-MEM (Gibco #31985-070) and then mixed 1:1 with Lipofectamine RNAiMAX reagent (Invitrogen #13778030) in Opti-MEM according to the manufacturer’s protocol. After a 5-minute incubation at room temperature, the siRNA-transfection reagent mixture was added to the culture medium at a final siRNA concentration of 17.5 pmoles per mL in a 24-well plate and 83 pmoles per well in a 6-well plate. At 24 h after transfection, cells were infected, and at the indicated time after infection, cells were processed for IFA or lysed for western blotting.

### Transcriptomic and gene ontology analysis

Paired-end (PE) RNA-seq reads were first aligned to human transcriptome annotations and genome assembly (hg38) using TopHat v2.1.1 (57, 58). Fragments per kilobase of transcript per million mapped reads (FPKM) values were calculated by Cufflinks v2.2.1 (59). Numbers of reads mapped to each gene were used to represent the gene expression values. Pairwise comparisons between infected and mock conditions were performed to detect differentially expressed (DE) genes using Cuffdiff (60). DE genes were defined as those with false discovery rate (FDR) less than 0.05. Up- and downregulated genes were defined using a fold change (FC) threshold (InfectedFPKM/MockFPKM) of 1.15, expressed as log2FC. The final visualization and exploration of results were carried out using the R-language package cummeRbund. Gene ontology (GO) analyses of biological process, cellular compartment, and molecular function genes were performed using the Database for Annotation, Visualization and Integrated Discovery (DAVID) v2021 (61, 62). Significant and enriched GO terms were obtained using DAVID, with a *P*-value cutoff of 0.05. The resultant findings were visualized using GraphPad Prism v8 for comprehensive representation.

### Statistical analysis

All biological experiments and assays were repeated three times. Values are shown as mean ± standard deviation (SD). Microsoft Excel or GraphPad Prism 9.5.1 software was used for statistical analysis. Significance was determined using a Student’s *t*-test. Asterisks were used to indicate statistically significant differences as in the figures and figure legends (*P <0.05; **P < 0.01; ***P < 0.001; ****P < 0.0001).

## Data Availability

The RNA-seq data generated in this study for A549 cells were deposited in the Gene Expression Omnibus (GEO) (http://www.ncbi.nlm.nih.gov/geo/) under accession number GSE310262. The RNAseq data for h-NPCs used in this study were deposited in GEO under accession number GSE309623.

## SUPPLEMENTAL MATERIAL

Table S1 jvi.02080-25-s0001.docx Viral reads in A549 cells.

Table S2 jvi.02080-25-s0002.docx A549 heatmap data.

Table S3 jvi.02080-25-s0003.docx A549 transcript levels.

Table S4 jvi.02080-25-s0004.docx Genes encoding proteins that can be ubiquitinated.

Table S5 jvi.02080-25-s0005.docx Viral reads in NPCs.

Table S6 jvi.02080-25-s0006.docx NPC heatmap data.

Table S7 jvi.02080-25-s0007.docx NPC transcript levels.

ASM does not own the copyrights to Supplemental Material that may be linked to, or accessed through, an article. The authors have granted ASM a non-exclusive, world-wide license to publish the Supplemental Material files. Please contact the corresponding author directly for reuse.
