# Supplementary material for: West Nile virus and Zika virus infections induce aggresome formation in human neural progenitor and A549 cells
Source: J Virol. 2026 May 11;100(6):e02080-25. doi: 10.1128/jvi.02080-25 (PMC13288479; doi:10.1128/jvi.02080-25)
Supplement: Table S1 — Viral reads in A549 cells. [file jvi.02080-25-s0001.docx]

**Supplementary Table 1. Viral read counts mapped from RNA-seq data obtained from infected A549 cells.**

| **Sample** | **#Reads** | **Log10(#Reads)** |
| --- | --- | --- |
| ZIKV 16h rep1 | 2,920 | 3.47 |
| ZIKV 16h rep2 | 2,788 | 3.45 |
| ZIKV 16h rep3 | 3,062 | 3.49 |
| ZIKV 32h rep1 | 16,036 | 4.20 |
| ZIKV 32h rep2 | 9,694 | 3.99 |
| ZIKV 32h rep3 | 8,390 | 3.92 |
| NY99 16h rep1 | 30,804 | 4.49 |
| NY99 16h rep2 | 31,356 | 4.50 |
| NY99 16h rep3 | 25,766 | 4.41 |
| NY99 32h rep1 | 45,308 | 4.66 |
| NY99 32h rep2 | 41,314 | 4.62 |
| NY99 32h rep3 | 47,648 | 4.68 |
