# Supplementary material for: West Nile virus and Zika virus infections induce aggresome formation in human neural progenitor and A549 cells
Source: J Virol. 2026 May 11;100(6):e02080-25. doi: 10.1128/jvi.02080-25 (PMC13288479; doi:10.1128/jvi.02080-25)
Supplement: Table S2 — A549 heatmap data. [file jvi.02080-25-s0002.docx]

**Supplementary Table 2. Log2FC transcript values of selected A549 UPR genes presented in the heatmap of Figure 2.**

| **Gene** | **ZIKV 16h** | **ZIKV 32h** | **NY99 16h** | **NY99 32h** |
| --- | --- | --- | --- | --- |
| ATF4 | 0 | 0 | 0.896021 | 0.966515 |
| ATF6 | 0 | 0.418113 | 0.270441 | 0.836728 |
| CHAC1 | 2.72031 | 1.87091 | 5.28822 | 4.21397 |
| CREBRF | 0 | 1.52322 | 1.84442 | 1.80106 |
| DDIT3 | 3.00514 | 3.69014 | 4.73085 | 4.86015 |
| DERL2 | 0 | 0.547304 | 0 | 0.277639 |
| DNAJB9 | 0.77491 | 1.3665 | 1.79331 | 1.67761 |
| EDEM1 | 0.688641 | 0.747262 | 0 | 0.276009 |
| EIF2AK3 | 0.708058 | 0.821736 | 1.03434 | 1.03858 |
| ERN1 | 1.28828 | 1.36539 | 1.98797 | 2.02364 |
| FICD | 1.02983 | 1.32672 | 1.36851 | 1.38736 |
| HERPUD1 | 1.51875 | 1.85726 | 1.69177 | 1.61475 |
| HSPA5 | 1.17528 | 1.23158 | 1.20083 | 1.50158 |
| MANF | 0.674666 | 1.04693 | 0.474847 | 0.467005 |
| SERP1 | 0.244632 | 0.584391 | 0.692735 | 0.604632 |
| XBP1 | 0 | 0 | 1.37534 | 1.75937 |
