# Supplementary material for: West Nile virus and Zika virus infections induce aggresome formation in human neural progenitor and A549 cells
Source: J Virol. 2026 May 11;100(6):e02080-25. doi: 10.1128/jvi.02080-25 (PMC13288479; doi:10.1128/jvi.02080-25)
Supplement: Table S3 — A549 transcript levels. [file jvi.02080-25-s0003.docx]

**Supplementary Table 3. Log2(FPKM+1) transcript values of selected A549 genes**

| **Gene** | **Mock 16h** | | | **ZIKV 16h** | | | **NY99 16h** | | |
| --- | --- | --- | --- | --- | --- | --- | --- | --- | --- |
|  | **Rep1** | **Rep2** | **Rep3** | **Rep1** | **Rep2** | **Rep3** | **Rep1** | **Rep2** | **Rep3** |
| HSPA5 | 6.59 | 6.57 | 6.55 | 7.78 | 7.75 | 7.68 | 7.77 | 7.79 | 7.55 |
| EIF2AK3 | 2.48 | 2.53 | 2.49 | 3.11 | 3.16 | 3.06 | 3.42 | 3.48 | 3.17 |
| HDAC6 | 2.66 | 2.73 | 2.69 | 2.94 | 3.07 | 3.04 | 3.09 | 3.16 | 3.30 |
| ATAT1 | 3.72 | 3.93 | 3.83 | 3.71 | 3.90 | 3.82 | 3.83 | 3.75 | 3.84 |
| VIM | 7.97 | 8.08 | 8.00 | 8.29 | 8.33 | 8.25 | 7.96 | 7.99 | 7.96 |
| UBE2L6 | 3.71 | 3.75 | 3.50 | 5.59 | 5.70 | 5.61 | 7.07 | 7.09 | 7.43 |
| **Gene** | **Mock 32h** | | | **ZIKV 32h** | | | **NY99 32h** | | |
|  | **Rep1** | **Rep2** | **Rep3** | **Rep1** | **Rep2** | **Rep3** | **Rep1** | **Rep2** | **Rep3** |
| HSPA5 | 6.76 | 6.77 | 6.74 | 7.93 | 7.99 | 8.03 | 8.32 | 8.20 | 8.17 |
| EIF2AK3 | 2.61 | 2.61 | 2.62 | 3.35 | 3.35 | 3.30 | 3.55 | 3.48 | 3.46 |
| HDAC6 | 2.90 | 3.16 | 3.13 | 2.80 | 3.02 | 3.30 | 3.24 | 3.37 | 3.21 |
| ATAT1 | 3.71 | 3.97 | 3.94 | 3.85 | 3.94 | 3.96 | 3.93 | 3.97 | 3.99 |
| VIM | 7.75 | 7.91 | 7.99 | 8.09 | 8.16 | 8.26 | 8.15 | 8.21 | 8.20 |
| UBE2L6 | 3.44 | 3.75 | 3.56 | 7.38 | 7.28 | 7.56 | 7.25 | 7.70 | 7.63 |
