# Supplementary material for: West Nile virus and Zika virus infections induce aggresome formation in human neural progenitor and A549 cells
Source: J Virol. 2026 May 11;100(6):e02080-25. doi: 10.1128/jvi.02080-25 (PMC13288479; doi:10.1128/jvi.02080-25)
Supplement: Table S4 — Genes encoding proteins that can be ubiquitinated. [file jvi.02080-25-s0004.docx]

**Supplementary Table 4. Number of significantly enriched genes in the Gene Ontology category “Ubiquitin-like (Ubl) protein conjugation” in infected A549 cells**

| **Sample** | **# genes annotated** | **p-value** | **-log10(p-value)** |
| --- | --- | --- | --- |
| ZIKV 16h | 430 | 5.99E-29 | 28.22 |
| ZIKV 32h | 444 | 5.57E-32 | 31.25 |
| NY99 16h | 915 | 2.28E-92 | 91.64 |
| NY99 32h | 965 | 2.50E-84 | 83.60 |
