# Supplementary material for: West Nile virus and Zika virus infections induce aggresome formation in human neural progenitor and A549 cells
Source: J Virol. 2026 May 11;100(6):e02080-25. doi: 10.1128/jvi.02080-25 (PMC13288479; doi:10.1128/jvi.02080-25)
Supplement: Table S5 — Viral reads in NPCs. [file jvi.02080-25-s0005.docx]

**Supplementary Table 5. Viral read counts mapped from RNA-seq data obtained from infected infected hNPCs.**

| **Sample** | **# of Reads** | **Log10 (# of Reads)** |
| --- | --- | --- |
| ZIKV 24h rep1 | 13,404 | 4.13 |
| ZIKV 24h rep2 | 13,576 | 4.13 |
| ZIKV 24h rep3 | 8,210 | 3.91 |
| ZIKV 48h rep1 | 66,260 | 4.82 |
| ZIKV 48h rep2 | 92,500 | 4.97 |
| ZIKV 48h rep3 | 58,708 | 4.77 |
| NY99 24h rep1 | 40,344 | 4.61 |
| NY99 24h rep2 | 38,576 | 4.59 |
| NY99 24h rep3 | 52,088 | 4.72 |
| NY99 48h rep1 | 37,242 | 4.57 |
| NY99 48h rep2 | 46,258 | 4.67 |
| NY99 48h rep3 | 37,924 | 4.58 |
