# Supplementary material for: West Nile virus and Zika virus infections induce aggresome formation in human neural progenitor and A549 cells
Source: J Virol. 2026 May 11;100(6):e02080-25. doi: 10.1128/jvi.02080-25 (PMC13288479; doi:10.1128/jvi.02080-25)
Supplement: Table S6 — NPC heatmap data. [file jvi.02080-25-s0006.docx]

**Supplementary Table 6. Log2FC transcript values of selected UPR genes from hNPCs presented in the heatmap of Figure 6B.**

| **Gene** | **ZIKV 24h** | **ZIKV 48h** | **NY99 24h** | **NY99 48h** |
| --- | --- | --- | --- | --- |
| ATF4 | 0 | 0 | 1.33569 | 1.03413 |
| ATF6 | 0 | 0 | 0 | 0 |
| CHAC1 | 1.80489 | 1.82138 | 3.88206 | 2.40159 |
| CREBRF | 0.621146 | 1.78189 | 1.1359 | 1.89359 |
| DDIT3 | 2.30461 | 2.97667 | 4.10765 | 4.02639 |
| DERL2 | 0.376526 | 0.34701 | 0.335006 | 0.313005 |
| DNAJB9 | 1.04798 | 0.988978 | 1.41774 | 1.05504 |
| EDEM1 | 0.466347 | 0.864975 | 0.567898 | 0.667273 |
| EIF2AK3 | 0.322314 | 0.84534 | 0.389216 | 0 |
| ERN1 | 0.671784 | 0.441407 | 0.896605 | 1.29317 |
| FICD | 0.894479 | 1.11861 | 1.55962 | 1.52148 |
| HERPUD1 | 1.60027 | 2.08228 | 1.98649 | 1.97793 |
| HSPA5 | 0.463432 | 0 | 0.426413 | 0.872006 |
| MANF | 0.457716 | 0.460619 | 0.30272 | 0.356017 |
| SERP1 | 0 | 0.388561 | 0.869692 | 0.338747 |
| XBP1 | 0.539917 | 1.20957 | 1.61871 | 1.96726 |
