# Supplementary material for: West Nile virus and Zika virus infections induce aggresome formation in human neural progenitor and A549 cells
Source: J Virol. 2026 May 11;100(6):e02080-25. doi: 10.1128/jvi.02080-25 (PMC13288479; doi:10.1128/jvi.02080-25)
Supplement: Table S7 — NPC transcript levels. [file jvi.02080-25-s0007.docx]

**Supplementary Table 7. Log2(FPKM+1) transcript values of selected genes from hNPCs.**

| **Gene** | **Mock 24h** | | | **ZIKV 24h** | | | **NY99 24h** | | |
| --- | --- | --- | --- | --- | --- | --- | --- | --- | --- |
|  | **Rep1** | **Rep2** | **Rep3** | **Rep1** | **Rep2** | **Rep3** | **Rep1** | **Rep2** | **Rep3** |
| HSPA5 | 7.93 | 8.01 | 8.00 | 8.50 | 8.40 | 8.42 | 8.45 | 8.27 | 8.50 |
| EIF2AK3 | 2.73 | 2.96 | 2.81 | 3.18 | 3.07 | 3.10 | 3.19 | 3.17 | 3.20 |
| HDAC6 | 4.24 | 4.21 | 4.27 | 4.11 | 4.33 | 4.49 | 4.44 | 4.64 | 4.55 |
| ATAT1 | 3.78 | 3.85 | 3.92 | 3.94 | 3.89 | 3.81 | 3.90 | 3.91 | 4.04 |
| VIM | 10.99 | 10.99 | 10.98 | 10.88 | 10.87 | 10.95 | 10.50 | 10.59 | 10.52 |
| UBE2L6 | 4.11 | 4.32 | 4.13 | 4.30 | 4.54 | 4.55 | 6.44 | 6.59 | 6.46 |
| **Gene** | **Mock 48h** | | | **ZIKV 48h** | | | **NY99 48h** | | |
|  | **Rep1** | **Rep2** | **Rep3** | **Rep1** | **Rep2** | **Rep3** | **Rep1** | **Rep2** | **Rep3** |
| HSPA5 | 8.14 | 8.04 | 7.94 | 8.11 | 8.17 | 8.21 | 8.91 | 8.93 | 8.86 |
| EIF2AK3 | 2.99 | 3.10 | 2.97 | 3.71 | 3.87 | 3.77 | 3.09 | 3.13 | 3.12 |
| HDAC6 | 4.08 | 4.21 | 4.24 | 4.47 | 4.43 | 4.52 | 4.39 | 4.54 | 4.68 |
| ATAT1 | 3.96 | 3.90 | 3.91 | 3.92 | 4.01 | 4.05 | 4.36 | 4.34 | 4.33 |
| VIM | 10.87 | 10.98 | 10.98 | 10.30 | 10.27 | 10.36 | 10.09 | 10.17 | 10.09 |
| UBE2L6 | 4.02 | 4.06 | 3.95 | 5.46 | 5.39 | 5.48 | 6.81 | 6.92 | 6.84 |
